# A compendium of 32,277 metagenome-assembled genomes and over 80 million genes from the early-life human gut microbiome

**DOI:** 10.1038/s41467-022-32805-z

**Published:** 2022-09-01

**Authors:** Shuqin Zeng, Dhrati Patangia, Alexandre Almeida, Zhemin Zhou, Dezhi Mu, R. Paul Ross, Catherine Stanton, Shaopu Wang

**Affiliations:** 1 Key Laboratory of Birth Defects and Related Diseases of Women and Children (Sichuan University), Ministry of Education, Department of Pediatrics, West China Second University Hospital, Sichuan University, Chengdu, China; 2 APC Microbiome Ireland, University College Cork, Cork, Ireland; 3 Teagasc Food Research Centre, Moorepark, Fermoy, Co. Cork, Ireland; 4 School of Microbiology, University College Cork, Cork, Ireland; 5 Department of Veterinary Medicine, University of Cambridge, Cambridge, UK; 6 European Bioinformatics Institute (EMBL–EBI), Wellcome Genome Campus, Hinxton, UK; 7 Pasteurien College, Medical College of Soochow University, Soochow University, Suzhou, China

**Keywords:** Metagenomics, Microbiome

## Abstract

Age-specific reference genomes of the human gut microbiome can provide higher resolution for metagenomic analyses including taxonomic classification, strain-level genomic investigation and functional characterization. We present the Early-Life Gut Genomes (ELGG) catalog with 32,277 genomes representing 2172 species from 6122 fecal metagenomes collected from children under 3 years old spanning delivery mode, gestational age, feeding pattern, and geography. The ELGG substantially expanded the phylogenetic diversity by 38% over the isolate microbial genomes, and the genomic landscape of the early-life microbiome by increasing recruitment of metagenomic reads to 82.8%. More than 60% of the ELGG species lack an isolate representative. The conspecific genomes of the most abundant species from children differed in gene diversity and functions compared to adults. The ELGG genomes encode over 80 million protein sequences, forming the Early-Life Gut Proteins (ELGP) catalog with over four million protein clusters, 29.5% of which lacked functional annotations. The ELGG and ELGP references provided new insights into the early-life human gut microbiome and will facilitate studies to understand the development and mechanisms of disturbances of the human gut microbiome in early life.

## Abstract (web summary)

Here the authors present a large-scale resource of the early-life human gut microbiome from children under three years old, which comprises 32,277 metagenome-assembled gut genomes, representing 2172 species, and more than 80 million gut proteins representing >4 million protein clusters, spanning multiple clinical factors including age, delivery mode, gestational age, and feeding patterns.

## Introduction

The human gut microbiome—the vast microbial ecosystem present in the gastrointestinal tract—has been suggested to play diverse and crucial roles in host health and various diseases throughout the course of life^1,2^. The acquisition and development of the gut microbiome in early life have long-lasting effects on the structure and function of this microbial community later in life^3^. Despite the increasing number of studies providing substantial insights into the early-life gut microbiome^4–9^, extensive genome-resolved metagenomic analyses of the early-life gut microbiome remain scarce. Having high-quality and extensive reference genomes of the early-life human gut microbiome can improve the resolution and accuracy of taxonomic and functional analyses, which is essential for driving future early-life microbiome studies.

Tremendous efforts have been undertaken to increase the number of isolate reference genomes from the human gut, such as the Human Microbiome Project (HMP)^10^, the Human Gastrointestinal Bacteria Genome Collection (HGG)^11^, and Culturable Genome Reference (CGR)^12^, however, the currently available reference genomes representing the human gut microbiome are still underrepresented^13,14^. Therefore, in parallel to culturing, de novo assembly of shotgun metagenomic reads and binning into metagenome-assembled genomes (MAGs)—a culturing-independent and reference-free approach—is thought to be a useful strategy to efficiently discover the potential microbial diversity that is recalcitrant to the current culturing approaches in the laboratory. Using MAGs has provided massive expansion of the tree of life from different environmental niches^15–17^. Studies have described some of the dynamic microbiome changes including taxonomic composition and strain-specific functional adaptation that occur during early life compared to adulthood^18^. For instance, most strains of *Bifidobacterium* that dominated the gut microbiome during breastfeeding and dissipated later in life typically carry high numbers of gene cluster responsible for human milk oligosaccharides (HMOs) utilization; whereas these gene cluster are no longer present in most bifidobacterial strains after weaning^19,20^. Understanding strain-specific differences in gene content and function also requires representative genomes of the early-life gut microbiome. Previous MAGs studies either analyzed samples exclusively from non-human gut source^21^, or from the human gut but with a relatively low proportion of early-life fecal samples^13,15^. Additionally, unifying the human gut genomes including MAGs and isolate genomes has indeed substantially provided novel insights into the richness, diversity and cultivability of gut microbiome at various taxonomic and functional levels^22^. However, there is currently no large-scale catalog of MAGs available specifically designed for the gut microbiome in early life.

Therefore, in order to fill this gap, we specifically analyzed 6122 fecal metagenomes from children under the first 3 years of life, and generated a set of 32,277 MAGs clustered into 2172 species-level clusters together with 86,678,654 genes representing 4,036,936 gene clusters, forming the Early-Life Gut Genomes (ELGG) and Proteins (ELGP) catalogs, respectively. With these comprehensive sequence collections, we characterized the taxonomic and functional profile of the early-life gut microbiome at the genome level and interrogated the genomic variations present in the gut microbiome of children associated with various clinical factors.

## Results

### Recovering 32,277 microbial genomes from over 6000 early-life gut metagenomes

**Fig. 1:**
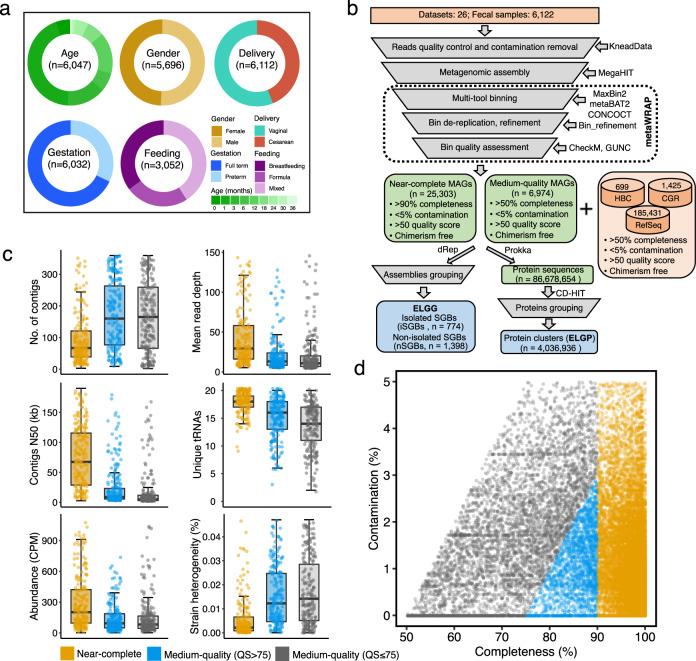
The reconstruction of sequence catalog from the early-life human gut microbiome. **a** The number and proportion of fecal metagenomes stratified by clinical features including age, gender, delivery mode, gestational age, and feeding patterns. **b** Overview of the computational pipeline to generate ELGG and ELGP catalogs. **c** Quality metrics across near-complete (*n* = 25,303), medium with quality score (QS) > 75 (*n* = 2063) and medium with QS ≤ 75 (*n* = 4911) MAGs. CPM copies per million reads. Boxes show the interquartile range (IQR), with the horizonal line as the median, the whiskers indicating the range of the data (up to 1.5× IQR), and points beyond the whiskers as outliers. **d** Completeness and contamination scores for each of 32,277 genomes. QS = completeness–5 × contamination.

To elucidate differences in the early-life gut microbiome at the genome level and also to expand the genomes for novel human gut lineages during early life, we employed a combination of metagenomic assembly and binning on 6122 multi-country distributed metagenomes across four continents from children from birth to three years old (Fig. 1a; Supplementary Data 1). Compared to the metagenomes that were used to build the Unified Human Gastrointestinal Genome (UHGG)^22^, 1904 metagenomes overlapped. The MAGs were produced by three different binning tools (i.e., MetaBAT^23^, MaxBin^24^, and CONCOCT^25^), and then integrated and refined to remove duplicates and improve the quality of assembled genomes with metaWRAP^26^ (Fig. 1b). Following this pipeline, a total of 42,054 MAGs were met or exceeded the medium-quality (≥50% completeness and <10% contamination) based on the “Minimum information about a metagenome-assembled genome” (MIMAG) standard^27^. In order to provide stricter genome quality control, we selected those genomes having completeness >50% and contamination <5% together with genome quality score (defined as completeness–5×contamination, QS) > 50 and free of chimerism (passed by GUNC^28^), resulting in 32,277 MAGs for subsequent analyses, which we referred to as the ELGG catalog (Fig. 1c, d; Supplementary Data 2). The median size of the 32,277 MAGs was 2.59 megabases (Mb) (interquartile range, IQR = 2.08–3.75 MB) with N50 values between 1.7 kilobases and 2.8 Mb. Among the ELGG catalog, 25,303 MAGs (accounting for 78.4% of the total dataset) were >90% complete (IQR = 97.3–99.7%) and <5% contaminated (IQR = 0.00–1.04%), hereafter referred to as ‘near-complete’ genomes. A subset of 4614 MAGs (18.2% of near-complete genomes) had 5 S, 16 S and 23 S rRNA genes as well as at least 18 of the standard tRNAs, which can be classified as the ‘high quality’ draft genomes based on the MIMAG standard^27^. The relatively low proportion of high quality recovered MAGs was comparable with previous large-scale studies of human gut MAGs^13,22^ due to the typical challenge in the MAGs assembled from metagenomes with short reads. The rest of the ELGG catalog consists of 6974 medium-quality MAGs (>50% completeness and <5% contamination) (Fig. 1d). The other genome statistics (including contig number and N50, genome depth, and relative abundance) supported the consistent high quality of near-complete MAGs compared to medium-quality MAGs even when the latter were stratified based on the QS at the threshold of 75 (Fig. 1c).

In line with previous studies^15,22^, the ELGG catalog was further investigated at the level of strain heterogeneity per genome by using CMSeq^15^, which has been suggested to represent a useful measure to assess genome quality. We found that the median strain heterogeneity (proportion of polymorphic positions) of genomes from the ELGG catalog was 0.005% (IQR = 0.001–0.031%; Fig. 1c), which is much lower than the UHGG catalog (0.06%) that included the human gut samples covering all ages^22^. The near-complete genomes displayed a lower level of strain heterogeneity compared to the medium-quality genomes from the ELGG catalog (Fig. 1c).

### A reference protein catalog for the gut microbiome early in life

**Fig. 2:**
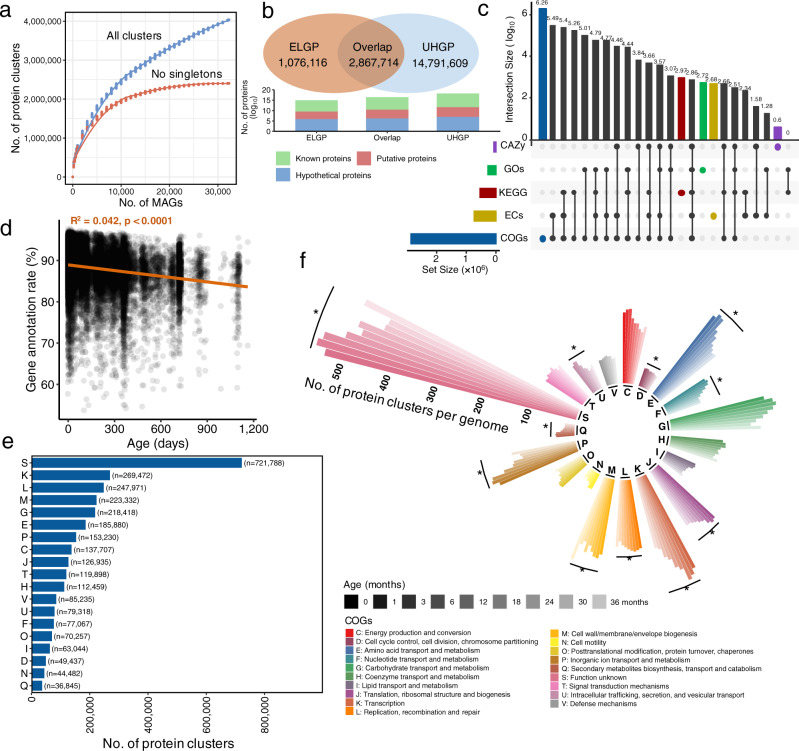
The ELGP catalog and functional characterization. **a** Rarefaction analysis of the number of protein clusters of early-life gut microbiome at 95% amino acid identity as a function of the number of genomes included. Curves are depicted for all the protein clusters and after excluding singleton protein clusters (containing only one protein sequence). **b** Overlap between the ELGP (orange) and UHGP (blue), both clustered at 95% amino acid identity. The bars at bottom indicate the number of proteins that the cluster representatives from three categories (ELGP exclusive, Overlap, and UHGP exclusive) encode, stratified as known, putative, and hypothetical proteins. **c** Number of proteins with functional annotation across the five functional categories and their degree of overlap. Vertical bars represent the number of proteins unique (color) to each functional category or shared (black) between the specific functional categories. Horizontal bars in the lower panel indicate the total number of proteins with functional annotation in each functional category. **d** Dynamics of the rate of protein characterization of ELGP along with the age of children. **e** COG functional annotation of the ELGP catalog clustered at 95% amino acid identity. Only functions with >5000 genes are plotted. **f** Dynamics of the rate of COG functional annotation of ELGP catalog clustered at 95% amino acid identity in response to the age of children. Vertical bars from left to right represent the age of children at 0, 1, 3, 6, 12, 18, 24, 30, and 36 months. Asterisk (*) indicates the significant difference (two-tailed Wilcoxon test, FDR < 0.05) between the rate of COG functional annotation of ELGP catalog at birth and 36 months old children.

To expand our understanding of the functions of early-life gut microbiome, the protein-coding sequences (CDS) for each of the 32,277 MAGs were predicted, resulting in a total of 86,678,654 genes. This accounted for 54.9% of all genes when taking the unbinned contigs from the 6122 metagenomic samples into account. After clustering the protein sequences at 95% amino acid identity, we obtained 4,036,936 protein clusters, forming the ELGP catalog. Rarefaction analysis indicated a saturation point was still not reached as the number of ELGP clusters steadily increased as a function of the number of MAGs included (Fig. 2a), and this pattern was also observed with the inclusion of all contigs from 6122 samples (Supplementary Fig. 1a), which was in line with pervious observations^22,29^. However, when removing protein clusters with one protein sequence, the number of protein clusters approached saturation (Fig. 2a; Supplementary Fig. 1a). This may suggest that although the microbial genes from children gut microbiome are still underestimated, the majority of undiscovered genes are likely to be rare. We further compared our early-life gene catalog to the large protein database—Unified Human Gastrointestinal Protein (UHGP)—that mainly includes microbial genes from the gut of adults and clustered at 95% protein identity (*n* = 20,239,340)^22^. This revealed that 2.9 million gene clusters from the ELGP overlapped with the UHGP catalog, but there was a large proportion (27.3%, *n* = 1,076,116) from the ELGP not represented in UHGP, and the total number of proteins from 1,076,116 clusters accounted for 5.4% when taking all 86,678,654 genes into consideration, underlying the uniqueness of the gut microbiome of children. Among those protein cluster representatives exclusively from ELGP or UHGP, 27.6% (*n* = 296,624) and 30.1% (*n* = 3,972,835) of representatives were respectively annotated with a known function, and the rest of the clusters were either putative or hypothetical proteins (Fig. 2b). Therefore, our results provide a comprehensive collection of the gut microbiome protein space early in life that may serve as a reference for early-life gut microbiome research.

To better elucidate the functional diversity of the early-life gut microbiome, we annotated gene functions of the ELGP catalog with currently available databases, including Clusters of Orthologous Genes (COGs), KEGG modules, level-4 Enzyme Commission categories (ECs), Gene Ontologies (GOs), and carbohydrate-active enzymes (CAZy). We found that a total of 70.5% of genes from the ELGP had a match to at least one of the databases of COGs (*n* = 2,844,021 genes across 24 functional categories), ECs (*n* = 722,946 genes matching 2658 enzymes), KEGG (*n* = 533,759 genes from 674 modules), GOs (*n* = 256,861 genes from 10,461 orthologous groups), and CAZy (*n* = 46,392 genes matching 104 families) (Fig. 2c). These results showed that a median of 88.7% (IQR = 85.9–91.0%) of genes per genome in the ELGG were annotated, and this rate was lower in genomes from children at 36 months of age (a median of 89.1% at birth vs. 86.5% at 36 months; linear model, *p* < 0.0001) (Fig. 2d). Based on the distribution of COGs functions that matched the largest number of ELGP genes, the most abundant genes with a known function present in the ELGP were involved in transcription, replication/recombination/repair, cell wall/membrane/envelope biogenesis, and carbohydrate transport and metabolism (Fig. 2e). The most highly represented families of ECs, KEGG, and GOs were DNA helicase (EC: 3.6.4.12), M00178 (ribosome, bacteria) and biological process (GO: 0008150). The predominant glycoside hydrolase family in the ELGP catalog was GH13, targeting the hydrolysis of a wide range of simple and complex glycans including di-, oligo-, and polysaccharides as well as related substrates, such as starch, amylose, and pullulan^30^ (Supplementary Fig. 1b). We again observed that the majority of the investigated COGs categories (11/19) were well-characterized at the first few months, and then gradually decreased as children aged (i.e., Wilcoxon test, FDR < 0.05, when compared to the annotated gene per genomes at birth to that from ≥36 months) (Fig. 2f).

### Early-life MAGs belonging to 2172 species-level clusters

**Fig. 3:**
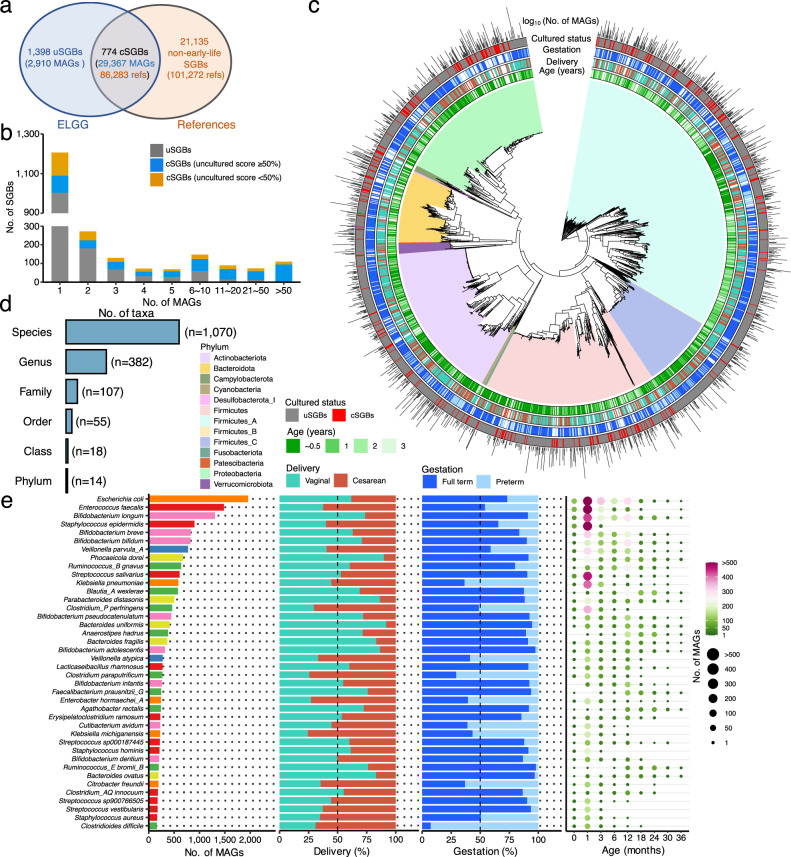
A total of 2172 species-level clusters (SGBs) obtained from 32,277 early-life MAGs. **a** Overlap of SGBs containing both MAGs and isolate reference genomes. SGBs containing MAGs and reference genomes are denoted as cultured SGBs (cSGBs), SGBs without reference genomes are denoted as uncultured SGBs (uSGBs), and those exclusively containing reference genomes are denoted as non-early-life SGBs. **b** The number of cSGBs and uSGBs as a function of the genome number within each SGBs. The uncultured score is calculated as the proportion of MAGs in the total genomes belonging to that SGB. **c** The phylogenetic tree of early-life gut microbiome built with 2171 bacterial representative genomes of the ELGG catalog. **d** The number of cultured taxa at different resolutions from 2172 representative genomes. **e** The number of MAGs in each SGBs, and only the top 40 most represented SGBs were displayed. The clinical factor (i.e., delivery mode, gestational age, and age) related to the MAGs per species are plotted.

To explore the number of culturable species that were included in the ELGG catalog, we clustered 32,277 MAGs together with 187,555 isolate reference genomes from NCBI RefSeq and two human gut culturing studies^11,12^. The species-level clusters (SGBs for species-level genome bins) were computed by using a multistep distance-based approach with at least 95% average nucleotide identity (ANI) and at least 30% overlap of alignment fraction (AF) (Methods). A total of 23,307 SGBs were generated, and the MAGs from the ELGG catalog were distributed into 2172 SGBs (Fig. 3; Supplementary Data 3). Among the 2172 SGBs, only 774 SGBs contained isolate reference genomes (denoted as cSGBs for cultured SGBs) containing 86,283 isolate reference genomes and 29,367 MAGs. A large proportion of 99.8% (*n* = 86,132) of 86,238 isolate reference genomes were near-complete (Supplementary Fig. 2). The other 1398 SGBs contained exclusively 2910 MAGs in total (denoted uSGBs for uncultured SGBs), indicating that 64.4% of the ELGG SGBs (9% of total MAGs) lack isolate genomes (Fig. 3a). When compared to the 4644 representatives of the UHGG using a distance cutoff of 0.05 (95% ANI), 13.4% of ELGG SGBs lacked a match to the UHGG. By counting the number of MAGs within each SGBs, it was observed that cSGBs represented the largest clusters, while uSGBs tended to be the rarest, with 1003 of uSGBs represented by a single genome, which was in line with the previous studies reconstructing MAGs from the environmental and host-associated microbiota^15,16,22^. Interestingly, cSGBs with >50% MAGs outnumbered uSGBs with 0–50% MAGs for clusters containing three or more genomes, underscoring the discovery power of large metagenomic cohorts (Fig. 3b). The early-life human microbial phylogenetic diversity of the 2171 bacterial SGBs was increased by 38% with the uSGBs, indicating the utility of these genomes to improve the classification of sequences from the early-life microbiome (Fig. 3c). The median pairwise distances of genomes within SGBs was 0.020 (IQR = 0.014–0.029) when including references and MAGs and 0.020 (IQR = 0.013–0.029) when only considering MAGs.

### Taxonomic landscape of the gut microbiome early in life

We further taxonomically annotated each species representative using the Genome Taxonomy Database Toolkit (GTDB-Tk) based on the GTDB database consisting of >311,000 bacterial and >6000 archaeal genomes that are comprised of isolate genomes, MAGs, and single-amplified genomes. We found that the ELGG catalog covered 14 known phyla (13 for bacteria and 1 for archaea), 18 known classes (17 for bacteria and 1 for archaea), 55 known orders (54 for bacteria and 1 for archaea), and 382 known genera (381 for bacteria and 1 for archaea) (Fig. 3d; Supplementary Data 3). Additionally, there were still 214 uSGBs including 339 MAGs that were not classified at the species level, indicating the lack of microbial representation in the current GTDB database. The top five uSGB classified genera were *Collinsella* (71 uSGBs with 143 MAGs), *Streptococcus* (33 uSGBs with 43 MAGs), *Haemophilus* D (13 uSGBs with 17 MAGs), *Veillonella* (13 uSGBs with 14 MAGs), and ﻿*Bifidobacterium* (9 uSGBs with 16 MAGs). Compared to the UHGG collection that is mainly comprised of microbial genomes from adults^22^, the phylum Firmicutes_A (705 SGBs with 7,765 MAGs in ELGG catalog) took up the largest proportion of SGBs in both children and adult gut microbiomes, followed by Firmicutes (390 SGBs, 7102 MAGs), Actinobacteriota (359 SGBs, 6188 MAGs), Proteobacteria (336 SGBs, 5409 MAGs), and Firmicutes_C (165 SGBs, 2,007 MAGs) (Supplementary Fig. 3a). All these top five phyla in children gut microbiome were represented by over 60% of uSGBs (Supplementary Fig. 3b). When compared at higher taxonomic resolution, a distinct difference was observed between children and adults gut microbiota. The MAGs assembled from children gut microbiome mainly consisted of the genus *Streptococcus* (164 SGBs, 2112 MAGs), *Collinsella* (129 SGBs, 534 MAGs), *Veillonella* (89 SGBs, 1501 MAGs), *Haemophilus* D (78 SGBs, 418 MAGs), and *Bifidobacterium* (58 SGBs, 4,604 MAGs) (Supplementary Fig. 3a); while the top genera from the UHGG catalog were *Collinsella*, *Prevotella*, *Streptococcus*, *Bacteroides*, and *Alistipes*.

At species level, the most represented SGBs in the ELGG catalog were *Escherichia coli*, *Enterococcus faecalis*, *Bifidobacterium longum*, *Staphylococcus epidermidis*, and *Bifidobacterium breve*, which completely differed from the genomes of the UHGG catalog (Fig. 3e). We further stratified the MAGs within each species according to delivery mode [vaginal and cesarean section (C-section)], gestational age (full-term and preterm) and the age of children at sampling. The MAGs belonging to species *E. faecalis*, *S. epidermidis*, *Clostridium* spp., *Veillonella* spp., *Klebsiella* spp., and *Streptococcus vestibularis* were mainly reconstructed from children born by C-section and/or preterm children. These species are potentially pathogenic and commonly associated with the hospital environment^4,31^. The majority of these MAGs were derived from fecal samples collected within the first year of life, highlighting the specificity of the ELGG catalog for the early-life gut microbiome. Notably, some MAGs were not reconstructed from the first few months after birth, but obtained at a later time, such as *Anaerostipes hadrus* and *Ruminococcus_E bromli_B*.

Rarefaction analysis of the total number of SGBs as a function of the number of MAGs indicated that the species from the ELGG catalog has not approached saturation, highlighting that more species remain to be discovered in the gut microbiome of children (Supplementary Fig. 3c). However, in line with the rarefaction analysis based on genomes from the UHGG catalog^22^, this unsaturated status was mainly attributed to rare members of the gut microbiota, as there were 1206 SGBs with only one MAG from the ELGG catalog (Supplementary Fig. 3d). When only considering SGBs containing at least two conspecific MAGs, the number of species was much closer to saturation (Supplementary Fig. 3c). When looking into the geographic prevalence of SGBs in each continent (i.e., Asia, Europe, North America, and Oceania), the most prevalent species worldwide included *﻿E. coli*, *B. longum*, and *E. faecalis* (Supplementary Fig. 4). Meanwhile, there were a number of SGBs with various rates of prevalence in each continent. For example, species of *Clostridium* spp., *Klebsiella michiganensis*, *Citrobacter freundii*, and *Clostridioides difficile* were more prevalent in the samples of North America, which may be attributable to the high proportion (77%) of fecal samples collected from preterm children.

### Comparison of SGBs across studies with the same metagenomic datasets

To investigate the reproducibility of SGBs from the ELGG catalog, we clustered the subset of MAGs with >50% genome completeness and <5% contamination and free of chimerism from a common set of 941 metagenomes from Bäckhed et al.^32^ and Vatanen et al.^33^ that were available in another two previous human gut MAG studies (i.e., Nayfach et al.^13^ and Pasolli et al.^15^) (Supplementary Data 4). Different assembly and binning approaches were applied in the three studies, i.e., Pasolli *et al*. assembled and binned with metaSPAdes and MetaBAT2; Nayfach et al. used MegaHIT and a combination of MaxBin2, MetaBAT2, CONCOCT and DAS Tool for assembling, binning and refinement. We observed that the pattern of MAG number produced from each sample was consistent across the three studies, but a slight increase (Wilcoxon test, *p* < 0.01) in the total number of MAGs was observed with our pipeline (*n* = 5203) compared to Nayfach et al. (*n* = 4284) and Pasolli et al. (*n* = 4728), respectively (Supplementary Fig. 5a). By calculating the proportion of shared SGBs on a per-sample basis with one other study (referred to as SGBs similarity, Methods), the median of SGBs similarity of the current study compared to the other two previous studies reached 100% for both Nayfach et al., and Pasolli et al. (Supplementary Fig. 5b). In addition, conspecific MAGs reconstructed from the same samples by different studies had a median ANI and AF of 99.9% and 93.9%, respectively (95.0% AF with near-complete MAGs and 85.3% AF with medium-quality MAGs; Supplementary Fig. 5c). These results suggest a high reproducibility of popular assembly and binning tools used in large-scale genome reconstructions, in line with previous comparisons^22^.

### Enlargement of the pan-genomes of key *Bifidobacterium* spp. early in life

**Fig. 4:**
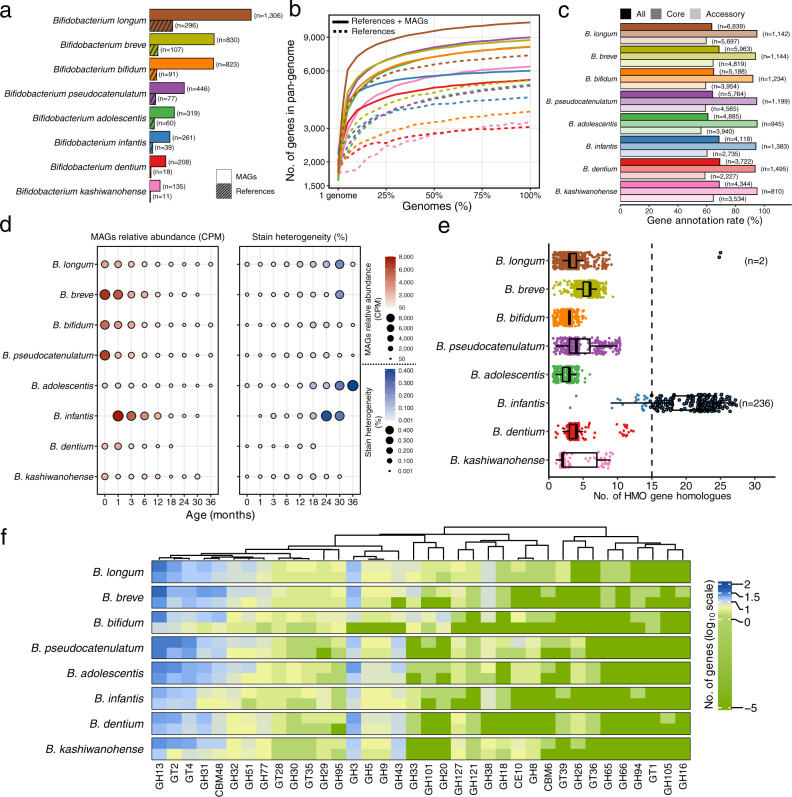
Characterization of key early-life *Bifidobacterium* spp. from ELGG catalog. **a** The number of genomes stratified by MAGs and reference genomes. **b** The pan-genome plot represented by the accumulated number of genes as a function of the number of genomes stratified by MAGs and reference genomes. **c** The rate of functional annotation across databases of COGs, KEGG, GOs, ECs, and CAZy for each species stratified by core and accessory genes. The number in parentheses indicates the number of genes with functional annotation. **d** Dynamics of the relative abundance and strain heterogeneity of MAGs in response to the age of children. **e** The number of gene homologs matched to a well-characterized gene cluster responsible for HMOs utilization from each species. Boxes show the interquartile range (IQR), with the vertical line as the median, the whiskers indicating the range of the data (up to 1.5× IQR), and points beyond the whiskers as outliers. **f** The glycobiome (columns) colored by the number of genes per genome (rows) of each species annotated with the CAZy database. The log_10_ scaled value (after adding a pseudocount of 1 × 10^−5^ to avoid non-finite values resulting from zero gene) is plotted.

*Bifidobacterium* represents the dominant genus in the gut microbiota of children and is known as the pioneering microbial member that influences microbiota succession and the capability of the host to utilize prebiotic HMOs early in life. A depletion of *Bifidobacterium* or their genes for the utilization of HMOs has recently been indicated to be involved in host systemic inflammation and immune imbalance^34^. Based on the GTDB annotation, we greatly expanded the diversity of *Bifidobacterium* intraspecies diversity by a range of 4 (*B. longum*) to 12 (*Bifidobacterium ﻿kashiwanohense*) times compared to the reference genomes belonging to the top eight *Bifidobacterium* SGBs that contained more than 100 MAGs. The largest SGB is *B. longum* with 296 reference genomes and 1306 added MAGs, followed by *B. breve* (107 reference genomes; 830 MAGs), *Bifidobacterium bifidum* (91 reference genomes; 823 MAGs), and *Bifidobacterium pseudocatenulatum* (77 reference genomes; 446 MAGs) (Fig. 4a). The pan-genome of each SGB is defined as the sum of the genes including core and accessory genes of all the genomes within that SGB^35^. The ELGG increased the size of the pan-genome per species up to a range of 5385 (*Bifidobacterium dentium* with 2337 exclusively from MAGs) to 10,759 (*B. longum* with 3522 exclusively from MAGs) that were higher than the reference genomes (Fig. 4b). This may indicate the large proportion of bifidobacterial metabolic functions that have not been uncovered based on current culturing approaches. By quantifying the abundance of these genomes in the metagenomic samples, we found that the relative abundance of bifidobacterial species decreased as children aged from birth to 3 years old (Fig. 4d). In addition, we found a lower level of strain heterogeneity in samples from early life (first 6 months), which may reflect the relatively simple dietary components (e.g., breastfeeding) in this period.

Next, we functionally annotated the pan-genomes of each *Bifidobacterium* species by mapping them against the broad range of databases including COGs, KEGG, GOs, ECs, and CAZy, and found that a proportion of genes between 30.9% (for *B. ﻿dentium*) and 39.2% (for *Bifidobacterium adolescentis*) lacked a match to any database. When we stratified the genes as core and accessory, the majority of unmatched genes were accessory (only a proportion of 56.0–64.6% genes matched), and over 92% of core genes were annotated (Fig. 4c). According to COG categories, the replication/recombination/repair, carbohydrate transport and metabolism, transcription, and amino acid transport and metabolism were the most prevalent known functions (Supplementary Fig. 6a). In addition, a total of 271 KEGG modules were encoded by the eight bifidobacterial species present in the ELGG (Supplementary Fig. 6b; Supplementary Data 5), with the main functions relating to multiple sugar transport system (M00207), ribosomal structure (M00178), putative ABC transport system (M00258), and raffinose/stachyose/melibiose transport system (M00196), reflecting their high capabilities of carbohydrate metabolism.

As the main microbial degraders of carbohydrates in the gastrointestinal tract early in life, we further profiled the glycobiome of bifidobacterial species based on the CAZy profiles (Fig. 4f). A total of 26 glycoside hydrolases (GH), 7 glycosyl transferases (GT), two carbohydrate-binding modules (CBM), and one carbohydrate esterase (CE) were observed across eight bifidobacterial species including reference genomes and MAGs. Notably, GH13 (followed by GT2, GT4, GH3, and GH31) were the most prevalent CAZy families within the bifidobacterial glycobiome, which has been proven to have the capacity to break down a wide range of carbohydrates dominant in the diet^30^. Compared to reference genomes, MAGs in the ELGG were annotated with higher and/or distinct gene families involved in carbohydrate metabolism. For instance, the MAGs from *B. bifidum* contain 27 CAZy families, 10 of which were not found in reference isolate genomes. The CAZy families present in MAGs but absent from reference isolate genomes included GH3, GH5, GH9, GH43, GH127, GH38, CE10, GH8, CBM6, and GH94. Considering breastfeeding during infancy, we further explored the functional potential of the MAGs in terms of HMO utilization by investigating the presence of gene cluster described as involved in HMO transport and degradation in *B. infantis* (ATCC 15967). MAGs from *B. longum* subspecies clade, *B. infantis*, carried a high number of HMO homologs, (236 out of 261 MAGs had at least 15 homologs, accounting for 50% of the HMO gene cluster) (Fig. 4e), while only two MAGs from *B. longum* carried gene cluster related to HMO metabolism, indicating the distinct capacity in HMO utilization of bifidobacterial species. When comparing the relative abundance of *B. infantis* with other B*. longum* genomes, a higher (Wilcoxon test, *p* < 0.0001) abundance of *B. infantis* was observed in all continents except for Oceania (Supplementary Fig. 6c), indicating the competitive advantage of *B. infantis* strains in early life that may be conferred by the presence of HMO gene cluster.

### Over 80% of early-life gut metagenomic sequences represented in the ELGG

To assess how representative the ELGG is as a genomic reference for metagenomes from the human gut in early life, we compared the mapping rate of 353 child fecal samples aged within the first 3 years against the ELGG catalog and another two large-scale reference collections, i.e., CIBIO (*n* = 4930)^15^ and UHGG (*n* = 4644)^22^. Using Bowtie2, we obtained a median mapping rate of 82.8% (IQR = 72.7–88.8%) with the ELGG catalog. This level of classification was higher than that obtained with the CIBIO and UHGG catalogs [69.5% (IQR = 61.1–76.4%) and 71.2% (IQR = 62.1–77.8%) respectively; Wilcoxon test, *p* < 0.0001] (Supplementary Fig. 7a; Supplementary Data 6). Additional evidence to support the specificity of the ELGG for classification of the early-life gut microbiome was the slightly lower mapping rate [a median of 66.7% (IQR = 60.2–73.2%, ELGG) compared to 72.1% (IQR = 69.2–75.0%, CIBIO) and 73.2% (IQR = 69.5–75.5%, UHGG); Wilcoxon test, *p* < 0.0001] when aligning metagenomic sequencing reads from the adult fecal samples (*n* = 510) against each catalog (Supplementary Fig. 7b; Supplementary Data 6).

### Conspecific genomic diversity associated with delivery mode

**Fig. 5:**
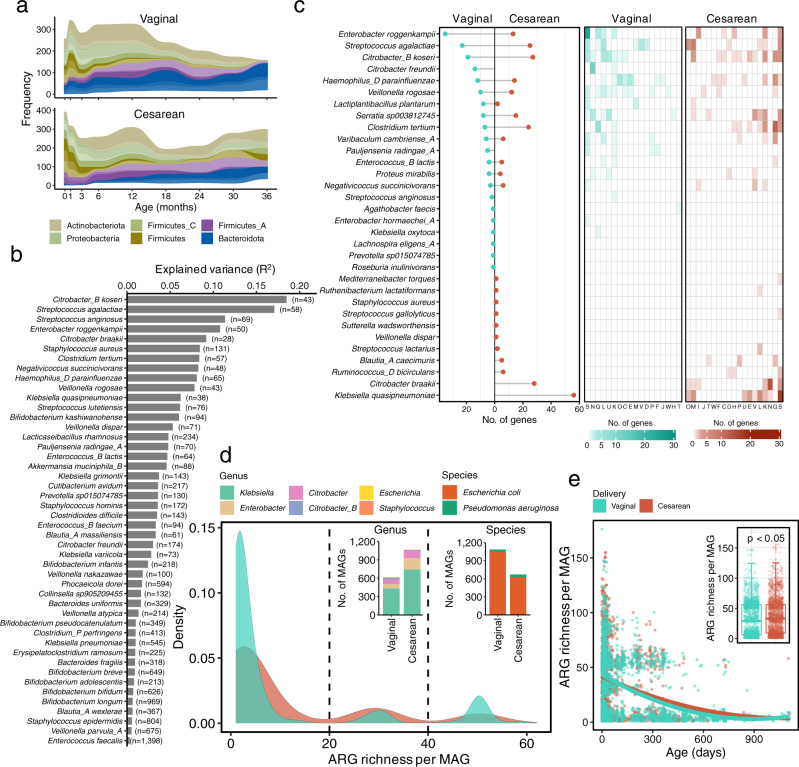
Influences of delivery mode on early-life gut microbiome at a genome-resolved level. **a** Prevalence of 16 bacterial genera in children stratified by delivery mode over time, where each genus was colored by its phylum. Only genera with >10% prevalence in children born by any of delivery modes are plotted. **b** The explained variance (R^2^) contributed by delivery mode of 46 species that were significantly (PERMANOVA, FDR < 0.05) associated with delivery mode based on the hamming distance of core genes per species. The number in parentheses indicate the number of MAGs of this species. **c** The number of genes that were prevalent in C-section born children or vaginally born children (>70% in C-section born children and <30% in vaginally born children, and vice versa) for each species and their associated functions annotated by COGs database. **d** The density of antibiotic resistance genes (ARGs) richness in each genome of ELGG, and the taxonomic assignment of the genomes at genus (left inset) and species (right inset) level. **e** The dynamics of ARGs richness from the early-life human gut microbiome in response to the age of children. The gut microbiome from children born by C-section carried higher (two-tailed Wilcoxon test, *p* < 0.05, inset) number of ARGs than that of children born vaginally. The inset boxes show the interquartile range (IQR), with the horizonal line as the median, the whiskers indicating the range of the data (up to 1.5× IQR), and points beyond the whiskers as outliers.

Children born by C-section display a significantly distinct gut microbial acquisition and development in the first few years compared to children born vaginally^4,6^, and several studies have attempted to restore the gut microbiota by probiotic supplements^36^, vaginal swabbing^37^, or fecal microbiota transplantation^38^ due to this disordered microbiome being positively linked with various diseases later in life^39^. We, therefore, leveraged the ELGG catalog together with the available metadata to address the taxonomic and functional differences associated with C-section at a genome level. A total of 18,836 and 13,412 MAGs were obtained from vaginally (*n* = 3299 samples) and C-section (*n* = 2612 samples) born children, respectively, with 1 to 38 MAGs per sample (mean ± SD: 5.71 ± 4.18) for the former and 1 to 27 MAGs per sample for the latter (5.13 ± 3.37) (Wilcoxon test, *p* < 0.0001). When adjusting by the sequencing depth, the number of MAGs per million paired reads differed (0.32 ± 0.35 for vaginal and 0.37 ± 0.24 for C-section; Wilcoxon test, *p* < 0.001) (Supplementary Fig. 8a). The majority of MAGs for either delivery mode were annotated as phyla of Firmicutes/_A/_C, ﻿Actinobacteriota, Proteobacteria, Bacteroidota, and ﻿Verrucomicrobiota (Supplementary Fig. 8b). When stratified by children’s age, the prevalence of the genera ﻿*Bacteroides*/*Phocaeicola* and *Parabacteroides* belonging to ﻿the Bacteroidota phylum present in C-section born children were at lower levels (Wilcoxon test blocked by children age, *p* < 0.05), while the genera *Veillonella* and *Klebsiella* were higher (Wilcoxon test blocked by children age, *p* = 0.035 and *p* = 0.056, respectively) than those born vaginally, in particular in the first few months of life (Fig. 5a). This observation confirms and expands the previous results obtained with the read-based analysis^4,40^.

Beyond the observed differential taxa, the reconstructed genomes enabled us to explore the intraspecies genetic and genomic diversity of the gut microbiome associated with delivery mode in early life. Only SGBs with at least 10 conspecific near-complete genomes (>90% completeness and <5% contamination) from both vaginal and C-section born children were considered in this part of the analysis. A total of 116 species were retained, covering the phyla Firmicutes/_A/_C (*n* = 30/34/7), Proteobacteria (*n* = 20), Actinobacteriota (*n* = 16), Bacteroidota (*n* = 7), and ﻿Verrucomicrobiota (*n* = 2), totaling 20,816 genomes (82% of all near-complete genomes of ELGG) (Supplementary Data 7). When looking into the intraspecies genomic diversity, the average pairwise genetic distances of core genes for each SGB was below 5% (typically used as a threshold to define bacterial species) (Supplementary Data 7). When setting the threshold of ANI at a higher level based on whole genomes, a number of subspecies from 1 to 88 and a range of 2 to 596 were obtained at a cutoff of 97% and 99%, respectively, suggesting the existence of diverse subspecies populations (Supplementary Data 7). We further sought to determine to what extent delivery mode contributed to these variances. The intraspecies variation within the core genomes of 46 species, and the genomic distances (based on gene presence/absence) of 64 species were significantly (PERMANOVA, FDR < 0.05) influenced by delivery mode with effect size up to 18.4% and 17.3%, respectively (Fig. 5b; Supplementary Fig. 8c). Notably, *Streptococcus agalactiae* also known as group B streptococci was highly sensitive to genetically associate with delivery mode.

The pan-genome size of the 116 species here analyzed ranged from 1788 (*Negativicoccus succinicivorans*, *n* = 58 genomes) to 25,698 (*Phocaeicola dorei*, *n* = 677 genomes) (Supplementary Data 7). A total of 31,976 unique genes across 116 species were observed with varying levels of prevalence among genomes from children born vaginally or via C-section. Functions encoded by the genes prevalent (>70%) in C-section born children but not children born vaginally (<30%) were mainly involved in carbohydrate transport/metabolism, cell motility, transcription, and cell wall/membrane/envelope biogenesis (Fig. 5c). The majority of differentially prevalent genes were not related to HMO degradation and utilization as only 4738 unique genes (out of 31,976) were matched with a HMO gene cluster from strain *B. infantis* ATCC 15697.

Mothers who give birth by C-section usually undergo antibiotic treatment, which may result in different antibiotic resistance profiles reflected in the gut microbiome of children. We thus functionally annotated the genomes with antibiotic resistance genes (ARGs) based on the Comprehensive Antibiotic Resistance Database (CARD). The average ARG richness per genome from C-section born children was higher (Wilcoxon test, *p* < 0.0001) than that of vaginally born children (11.6 vs. 10.0 type of ARGs), however, both distributions of ARG richness of genomes from either delivery mode were clearly trimodal (Fig. 5d), with a larger peak at only one ARG, and the other two smaller peaks at 31 and 50 genes, respectively. The origins of ARGs within each peak differed among children born by different delivery modes. In the second peak, genera *Klebsiella*, *Enterobacter*, and *Citrobacter* were the main contributors in children born by C-section; while the third peak was mainly contributed by *E. coli* that was more prevalent in vaginally born children. Apart from *E. coli*, 73 MAGs from *﻿Pseudomonas aeruginosa* were found to carry higher (Wilcoxon test, *p* < 0.0001) richness of ARGs (58.8 ± 1.38) than *E. coli* (50.2 ± 2.27). Among these 73 MAGs, 68 were reconstructed from preterm children within the first 6 months (62 genomes within the first month). As children aged, the richness of ARGs in the gut microbiome generally decreased, from an average of 42.6 at one month to 6.8 ARGs at over 36 months old (Fig. 5e). Notably, the richness of ARGs present in the gut microbiomes of children born by C-section was overall higher than that of vaginally born children (an average of 36.9 vs. 32.5 AGRs; Wilcoxon test, *p* < 0.0001). When comparing the genomes within the same species from children born differently in terms of ARG richness, 15 species showed differential ARG richness, and 12 species contained higher numbers (Wilcoxon test, *p* < 0.05) of ARGs in C-section born children than those born vaginally, while three species (*﻿﻿﻿Pauljensenia radingae_A*, *Clostridium paraputrificum*, and *﻿Clostridium_P perfringens*) exhibited opposite patterns (Supplementary Fig. 8d). The most common mechanisms of antibiotic resistance discovered in the 20,816 genomes included antibiotic efflux, antibiotic target alteration, and antibiotic inactivation (Supplementary Fig. 8e).

### Comparisons of gut microbiome between children and adults

**Fig. 6:**
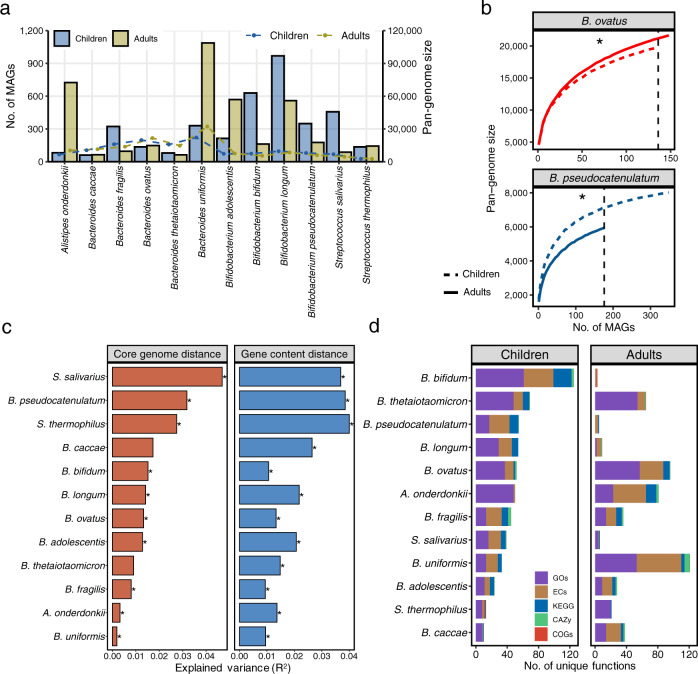
Comparisons of gut microbiome between children and adults. **a** Number of genomes (bar plot) and pan-genome size of each species from children and adults. **b** Pan-genome plot represented by the accumulated number of genes against the number of genomes of *B. ovatus* and *B. pesudocatenulatum* stratified by children and adults (two-tailed Wilcoxon test, *FDR < 0.05). **c** The explained variance (R^2^) contributed by age (children and adults) based on the hamming distance of core genes per species and Jaccard distance of presence/absence genes (two-tailed Wilcoxon test, *FDR < 0.05). **d** The unique functional annotations belonging to either children or adults categorized by databases of COGs, KEGG, GOs, ECs, and CAZy.

The comprehensive catalog of the early-life microbiome enabled us to explore the taxonomic and functional differences between the children and adult gut microbiomes at a genome level. We thus compared the five most represented genera in children (≤3 years) and adults (≥18 years) (i.e., *Alistipes*, *Bacteroides*, *Bifidobacterium*, *Prevotella*, *Streptococcus*, *Veillonella*) based on ELGG and UHGG catalogs^22^, totaling 12 species with >60 near-complete genomes (>90% completeness and <5% contamination). The pan-genome size was positively associated with the number of included genomes, but none of the species reached a plateau, even *Bacteroides ﻿uniformis* with the highest number of genomes (*n* = 1087) containing 32,215 genes in adults. Species ﻿of *Streptococcus thermophilus* had the lowest pan-genome size with 2572 for adults and 2639 for children from 143 and 136 genomes, respectively (Fig. 6a). This suggests additional genomes from each species remain to be discovered across populations. In the genus *Bacteroides*, genomes from adults contained a higher number of unique genes than those from children when considering the same number of genomes (Wilcoxon test, FDR < 0.05). In contrast, gene numbers of *﻿﻿Alistipes onderdonkii*, *B. adolescentis*, *B. longum*, *B. ﻿pseudocatenulatum*, and *Streptococcus salivarius* were higher (Wilcoxon test, FDR < 0.05) in children (Fig. 6b; Supplementary Fig. 9).

Notably, when looking into gene diversity (estimated by the Jaccard distance based on the presence/absence of genes per genome), genomes from adults showed higher (Wilcoxon test, FDR < 0.05) gene diversity on average than that of children for 5 out of 12 species, including *Bacteroides fragilis*, *Bacteroides ovatus*, *B. bifidum*, *S. salivarius*, and *S. thermophilus* (Supplementary Fig. 10a). These results indicate that genomes within these species in early life are more conserved, and more specific genes are acquired by the microorganisms later in life. On the contrary, the enriched species *B. longum* showed higher (FDR < 0.05) gene diversity than that of adults. We also explored the effect size and significance of age (≤3 years for children and ≥18 years for adults) on the gene diversity of each species. The results showed the distinct contribution of age (PERMANOVA, FDR < 0.05) to the genetic variation of species between children and adults. *S. salivarius* (R^2^ = 0.047 for hamming distance and R^2^ = 0.037 for Jaccard distance), *B. pseudocatenulatum* (R^2^ = 0.032; R^2^ = 0.039), and *S. thermophilus* (R^2^ = 0.027; R^2^ = 0.040) were the species most significantly associated with age (Fig. 6c).

Based on the multiple functional annotation schemes as ELGP, the pan-genome of species showed comparable rates of gene annotations between children and adults, but differed across species, namely, *B. ﻿adolescentis* with the lowest rates of 62.4% and 64.6% for children and adults respectively, and *S. thermophilus* with the highest respective rates of 84.6% and 85.3% for children and adults (Supplementary Fig. 10b). Based on the CAZy annotation of the pan-genomes, we found that gut microorganisms from children harbored a higher (Wilcoxon test, FDR < 0.05) number of specific CAZy families, most notably GH13, GT4, GT2, GH43, and GH3 (Supplementary Fig. 10c). Additionally, we sought to determine the functions that were unique to either children or adults. We found a large number of EC families among species (*n* = 1–38 in children and 0–57 in adults), KEGG modules (*n* = 0–23 in children and 0–13 in adults), CAZy families (*n* = 0–4 in children and 0–7 in adults), COGs (*n* = 0–1 for both children and adults) and GOs categories (*n* = 8–61 in children and 0–57 adults) to be specific to either children or adults (Fig. 6d; Supplementary Data 8). Within EC families of *B. bifidum*, the enzymes of GlfT2 (﻿EC 2.4.1.288; *n* = 27 genomes), asparagine synthase (EC 6.3.5.4; 17 genomes), and D-xylulose reductase (EC 1.1.1.9; 12 genomes) were the most prevalent in children; and the top families of CAZy from children included GH127 (5 genomes), GH94 (4 genomes), and CBM6 (1 genomes). Within EC families of *B. uniformis*, the enzymes of dTDP-6-deoxy-L-talose 4-dehydrogenase (﻿EC 1.1.1.34; 13 genomes), thiamine kinase (EC 2.7.1.89; 13 genomes), and histidine decarboxylase (EC 4.1.1.22; 13 genomes) were the most prevalent in adults; and the top families of CAZy from adults included PL4 (7 genomes), PL11 (3 genomes), AA10 (2 genomes), and CBM73 (2 genomes).

## Discussion

We provide a large-scale sequence resource of the human gut microbiome in early life, representing 32,277 newly reconstructed genomes and over 80 million protein sequences, and spanning multiple clinical factors including age, delivery mode, gestational age, and feeding patterns with significant influences on the early-life gut microbiome^4,7,19,32^. The ELGG catalog considerably expands the phylogenetic diversity of the early-life gut microbiome by increasing the classification rate of metagenomic sequencing reads (over 82%) and uncovering genomes without a cultured representative. Although the gut microbiome community is relatively simple in early life compared to adults, over 64% of the 2172 species-level clusters from ELGG lack a cultured representative, suggesting the importance and need to experimentally isolate and characterize the gut microbiome from children. Having this ELGG catalog can serve as a reference for future studies specifically for early-life gut microbiome research and help prioritize targets for experimental isolation and cultivation. Characterizing newly isolate strains from children may aid in the development of dietary supplements to help restore the gut microbiome composition perturbed by extrinsic or intrinsic factors, such as C-section delivery and antibiotic intervention.

With the establishment of the ELGG catalog, the strain dynamics, pan-genome and genomic diversity of the main *Bifidobacterium* species from the human gut in early life have been investigated at a genome level. The pan-genome of these bifidobacterial species has been greatly expanded by up to 12 times in comparison to the reference genomes only, substantially contributing to the genomic landscape of bifidobacterial species. However, nearly 40% of the pan-genome across bifidobacterial species remains uncharacterized functionally. Given significant influences of delivery mode on the development of the early-life gut microbiome, we further compared the gut microbiome from children born by C-section or vaginally at multiple levels of resolution and identified a set of species and genomic variations linked to delivery mode. Notably, the richness of ARGs gradually decreased as children aged, and microbial genomes from C-section-born children carried higher ARGs than that from children born vaginally. We speculate this pattern could be related either with the antibiotic treatment typically administered to mothers undergoing C-sections or with antibiotic interventions in children after birth. Moving from early life to adulthood, it has been known that along with changes in diet, physiological functions, and the immune system, the gut microbiome gradually approaches maturity as children grow^3,19^. Consistent with the initial hypothesis of the existence of age-specific microbial communities with distinct genomic and functional patterns^18,32^, we found that some microbial genomes from adults possessed higher gene diversity, suggesting that gut microorganisms in adults gain unique genes later in life. The genomes from either children or adults showed comparable rates of functional annotations across species, however, the most prevalent functions differed between children and adults, highlighting the importance of developing age-specific reference genomes targeted at particular populations.

Currently, linkages between the early-life human gut microbiome and health have been well recognized, and enhanced resolution and accuracy of taxonomic classification and microbial genomic adaptation will require more culture-based and bioinformatic work targeting specific periods of life or clinical contexts. Our newly reconstructed genomes represent a key step for the early-life human gut microbiome and provide new insights into the taxonomic, functional and genomic diversity of this period of life. The establishment of ELGG and ELGP catalogs will substantially improve our ability to understand the development and mechanisms of disturbances of the early-life gut microbiome. Our knowledge of the human gut microbiome composition and function in early life will have a profound impact on promoting or maintaining human health throughout the course of life.

## Methods

### Publicly available early-life gut metagenomic datasets and quality control

A total of 6122 paired-end sequencing runs collected by 26 studies for early-life gut metagenomes were downloaded from the NCBI SRA according to the accession numbers published in each included study. FASTQ files were retrieved by using fastq-dump v2.9.1 with the option “-split-3” from SRA Toolkit v2.9.1. These samples were distributed among 11 countries across four continents, with the United States, United Kingdom, and New Zealand being the top three represented. All metagenomic sequencing data were quality controlled and human contamination (hg19 human reference genome) was filtered by using KneadData v0.7.2 with default parameters, resulting in 1.3 × 10^11^ paired reads (87% of the raw sequencing reads).

### Metagenomic assembly and contig binning

The quality-filtered sequencing reads from each of 6122 metagenomes were assembled using MegaHIT v1.1.3^41^ with option “-min-contig-len 1000”, which resulted in 29,912,553 contigs, with a total length of 1.71 × 10^11 ^bp, and an average of N50 of 46,532 bp, calculated with the “stats.sh” script (format = 5) from BBMap v38.22 (https://sourceforge.net/projects/bbmap/). Depth of coverage of each contig was calculated by mapping the raw reads back to their assemblies using BWA-MEM v0.7.17^42^ with default options and then calculating the corresponding contig depth with SAMtools v1.10 and jgi_summarize_bam_contig_depths function from MetaBAT v2.12.1^23^. The gut MAGs from children were generated per sequencing run using three metagenomic binning tools (MetaBAT v2.12.1, MaxBin v2.2.6^24^, and CONCOCT v1.0.0^25^) using metaWRAP v1.3.1^26^ with default parameters. The minimal contig size for binning was set as default with 1000 bp for further processing, except for MetaBAT2, which required at least 1500 bp. Afterwards, the produced bins from each binning tool were integrated and refined with Bin_refinement module of metaWRAP with options “-c 50 -x 10”, corresponding to the criterion of medium-quality draft MAGs^27^. The quality (estimated completeness and contamination) of bins was evaluated with CheckM v1.0.12 lineage workflow^43^, implemented in metaWRAP with option “-quick”. In order to control the genome quality strictly, the resulting 42,054 MAGs were checked by GUNC v1.0.5^28^ to filter genomes potentially containing chimerism based on ‘pass.GUNC’ in the output file. The genome quality score was calculated as: completeness–5 × contamination. The ribosomal RNA (rRNA) genes were searched using Barrnap v0.9^44^ with options “--reject 0.01 --evalue 1e-03”, and transfer RNAs (tRNAs) of the standard 20 amino acids were identified with tRNAScan-SE v2.0.6^45^ with options “-A -Q” for archaeal species and “-B -Q” for species belonging to bacterial lineages based on the annotation of GTDB.

### Assessing the strain heterogeneity of MAGs

The CMSeq tool v1.0.3 was used to investigate the strain-level heterogeneity within each MAG^15^. Firstly, metagenomic reads from each sample used to generate MAGs were aligned to the assembled contigs using BWA-MEM, and the aligned files were sorted and indexed with SAMtools. Secondly, the protein-coding genes of the assembled contigs were predicted with Prodigal v2.6.3 implemented in the Prokka v1.14.6 with the default settings, and the resulting GFF file was used for subsequent analysis. Finally, the “polymut.py” script from the CMSeq package was used to detect the nonsynonymous mutations from those positions mapped with the PHRED quality score of at least 30 and a depth of coverage of at least 10x. A position was considered non-polymorphic if the dominant allele frequency was >80%. The level of strain heterogeneity of each MAG was calculated by the proportion of the total number of nonsynonymous mutations in the total number of considered positions.

### Reference genomes from public databases

The NCBI RefSeq database (accessed on 25th September 2020) that contains bacterial genomes and archaeal genomes were downloaded (https://ftp.ncbi.nlm.nih.gov/genomes/refseq/). In addition, the isolate genomes from the Human Gastrointestinal Bacteria Culture Collection (HBC, http://ftp.ebi.ac.uk/pub/databases/metagenomics/genome_sets/hbc_genomes.tar.gz)^11^, and Culturable Genome Reference (CGR)^12^ were also gathered. The completeness and contamination of all reference genomes were evaluated using CheckM lineage workflow with default settings. The genomes not having >50% completeness, <5% contamination, genome quality score <50, and failing to pass the chimerism detection by GUNC were then discarded, resulting in a total of 187,555 reference genomes including 184,392 bacterial genomes and 1039 archaeal genomes from RefSeq, 699 genomes from HBC, and 1425 genomes from CGR.

### Species-level clustering of reconstructed MAGs and reference genomes

The total set of 219,832 genomes (32,277 MAGs and 187,555 reference genomes) were clustered at an estimated species level (ANI ≥ 95%; refereed as SGBs)^22^ using dRep v2.6.2^46^ with the following options: “-pa 0.9 -sa 0.95 -nc 0.30 -cm larger, -S_algorithm fastANI v1.33”. In order to increase the computational efficiency to cluster the complete genome set, an iterative approach was applied where random chunks of 30,000 genomes were clustered independently, and then the selected representatives per cluster from each chunk were combined and subsequently clustered^22^. The genome with the higher score calculated based on the following formula was selected as the species representative in each iteration:

$${{{{{\rm{Score}}}}}}={{{{{\rm{CMP}}}}}}-5\times {{{{{\rm{CNT}}}}}}+0.5\times {{{\log }}}_{10}({{{{{\rm{N}}}}}}50)$$

where CMP and CNT represent the estimated completeness and contamination, respectively; and N50 is the minimum contig length in which 50% of the total genome is covered. In case of clusters that contained reference genomes and MAGs, the reference genomes were prioritized over MAGs and selected as the representative.

In addition, the pairwise distances for all conspecific genomes were calculated by using Mash v2.3^47^ with default sketch size. Afterwards, the phylogenetic tree of each species was built with the “complete” hierarchical clustering method from ‘fastcluster’ R package^48^, and then the number of sub-clusters were further obtained by setting a distance cutoff of 0.03 (97% ANI) and 0.01 (99% ANI) to investigate the within-species population diversity. The pairwise genome distances between 2172 representatives of ELGG and 4644 representatives of UHGG were calculated by Mash with default sketch size.

The obtained SGBs were subdivided into 3 groups: (i) cultured SGBs (cSGBs) containing at least one MAG and one reference genome (the uncultured score for a SGB was calculated as the proportion of MAGs in the total genomes belonging to that SGB); (ii) uncultured SGBs (uSGBs) containing exclusively MAGs; (iii) non-early-life SGBs that contained exclusively reference genomes.

### Evaluation of MAGs reconstrued across metagenomic datasets

The MAGs reconstructed from a common set of 941 metagenomes that contained MAGs with >50% completeness and <5% contamination and without chimerism detection by GUNC from the current and two previous human gut MAG studies^13,15^ were compared in terms of genomic and taxonomic features. The genome quality (completeness and contamination) from another two studies were re-estimated with CheckM lineage workflow with “-reduced_tree”^43^ in line with the option “-quick” in metaWRAP and GUNC for chimerism detection. The resulted genomes were clustered to SGBs using dRep with “-pa 0.9 -sa 0.95 -nc 0.30 -cm larger, -S_algorithm fastANI”. The similarity of SGBs of the same sample but in different studies was calculated as the percentage of shared SGBs in the smaller SGBs of the two studies. Conspecific genomes recovered in the same metagenomic samples but in different studies were also compared with the alignment fraction (AF) and ANI that were obtained from the output of dRep. Both the maximum AF and ANI for each pairwise comparison were considered.

### Taxonomic annotation and phylogenetic analysis

Taxonomic annotation of each species representative was performed with GTDB-Tk v2.1.0 (reference database version R207) with “classify_wf” workflow using default parameters^49,50^. The NCBI taxonomy annotation was also generated for each species representative using the “gtdb_to_ncbi_majority_vote.py” script available in the GTDB-Tk repository.

The phylogenetic tree of 2171 bacterial representative genomes of SGBs was built using FastTree v2.1.11^51^ with default settings using the protein sequence alignments generated by GTDB-Tk.

To estimate the increase in phylogenetic diversity (PD) contributed by the ELGG catalog, we computed the sum of branch length of the whole bacterial trees (PD_all_) and the sum of branch length from 773 cSGBs (PD_cSGBs_) implemented through the function ‘pd’ in the “picante” package^52^. The percentage gain in PD contributed exclusively by uSGBs was calculated as: 100 × (PD_all_ − PD_cSGBs_)/ PD_cSGBs_.

### Metagenomic read mapping

An additional number of 353 metagenomes with >50,000 sequencing paired reads that were randomly selected from child fecal samples but not used to generate ELGG or ELGP catologs (Supplementary Data 6) from five studies as cross-validation samples were retrieved from NCBI SRA with SRA Toolkit v2.11.2. These metagenomes were quality-filtered and host decontaminated (hg19 human reference genome) using KneadData with default parameters. Apart from the database reconstructed in the current study (ELGG, *n* = 2172), we built another two databases based on the representative genomes of ref. 15 (CIBIO, *n* = 4930) and the UHGG^22^ (*n* = 4644) with Bowtie2 v2.4.5^53^. Moreover, 510 metagenomes from adult fecal samples with >50,000 sequencing paired reads from five studies were downloaded from NCBI SRA v2.11.2. All downloaded metagenomes were aligned to these three databases respectively using Bowtie2 in “end-to-end” mode with the option “--very-sensitive”, and the generated mapping files were further filtered by removing alignments with an alignment score (AS:i tag) less than −20 that were likely to be spurious alignments in order to increase the reliability of mapping assessment.

### Comparisons between early-life and adult gut microbiome

The UHGG collection was used for analysing assembled genomes from adults (≥18 years), mostly comprising non-redundant genomes from three large-scale human gut studies^13,15,54^. The age information was retrieved from each of the three studies. The species from the five most prevalent genera in either the UHGG or the ELGG catalog and containing >60 genomes (>90% completeness and <5% contamination, as well as free of chimerism detected by GUNC) were selected for downstream analyses. The quality and the taxonomic annotation of genomes from UHGG was re-estimated with CheckM and GTDB-Tk, respectively, as performed for the ELGG. Finally, 12 species were retained for further analysis, i.e., *A. onderdonkii* (ELGG vs. UHGG: 82 vs. 725 MAGs), *Bacteroides caccae* (61 vs. 64 MAGs), *B. fragilis* (322 vs. 96 MAGs), *B. ovatus* (136 vs. 148 MAGs), *Bacteroides thetaiotaomicron* (80 vs. 63 MAGs), *B. uniformis* (330 vs. 1087 MAGs), *B. adolescentis* (213 vs. 569 MAGs), *B. bifidum* (629 vs. 162 MAGs), *B. longum* (969 vs. 559 MAGs), *B. pseudocatenulatum* (349 vs. 176 MAGs), *S. salivarius* (457 vs. 88 MAGs), *S. thermophilus* (136 vs. 143 MAGs). The corresponding genomes were then downloaded from the MGnify FTP site (http://ftp.ebi.ac.uk/pub/databases/metagenomics/mgnify_genomes/).

### Gene diversity, pan-genome analysis and functional annotations

#### Protein clusters

The protein-coding sequences (CDS) of genomes were predicted and annotated with Prokka, which employed Prodigal with options “-c” (protein predictions with closed ends only), “-m” and “-p single”. The 4,036,936 nonredundant ELGP clusters were generated from the set of 86,678,654 genes of 32,277 genomes by using CD-HIT v4.8.1^55^ at 95% protein identity (-c 0.95 -n 5 -M 0 -d 0 -g 1). Comparisons between ELGP and UHGP were conducted by clustering the 4,036,936 nonredundant ELGP and 20,239,340 UHGP-95 clusters with CD-HIT at 95% protein identity.

#### Pan-genome analysis

The pan-genome of each species was analysed by Panaroo v1.2.10^56^ with parameters “-c 0.90” for a minimum amino-acid identity of 90% for a positive match, “--core_threshold 0.90” and a family threshold (-f) of 50%, as well as option “--clean-mode strict” and “--merge_paralogs” by taking the GFF3 files created by Prokka. The core genes were defined as present in at least 90% of genomes. A single representative nucleotide sequence from each of the clusters was then annotated functionally by eggNOG-mapper v2.1.7 with database v5.0.2^57,58^. The COG^59^, KEGG module, CAZy, GO, and EC annotations were derived from the eggNOG-mapper results.

#### Antimicrobial resistance genes

Predicted genes (the representative nucleotide sequence from each of clusters producd by Panaroo) were annotated with The Comprehensive Antibiotic Resistance Database (CARD v3.2.3) by using its accompanying Resistance Gene Identifier (RGI, v5.2.0) with default parameters^60^, and only “Strict” and “Perfect” hits from RGI were retained for further analysis.

#### HMO gene homology analysis

The protein sequences of HMO gene cluster were obtained from strain *Bifidobacterium longum subsp. infantis* ATCC 15697 (GCF_000020425.1; BLON_RS12070-BLON_RS12215) from NCBI RefSeq database. Prediction of HMO gene cluster was performed by comparing the HMO protein sequences to the representative nucleotide sequence of each cluster in the pan-genome of a species from Panaroo using a tBLASTn v2.9.0 search. Specifically, identified hits were further filtered with identify percentage ≥70% and e-value <1e-10, and only the best hit per gene cluster was chosen.

### Statistical analysis

When evaluating the dynamics of microbial features, we stratified the continuous age of children into nine categories, namely 0 month (0−1 day, *n* = 326), 1 month (2−30 days, *n* = 2640), 3 months (31−90 days, *n* = 896), 6 months (91−180 days, *n* = 424), 12 months (181−360 days, *n* = 942), 18 months (361−540 days, *n* = 390), 24 months (541−720 days, *n* = 309), 30 months (721−900 days, *n* = 76), and 36 months (900−1,162 days, *n* = 44).

Statistical significance was verified through Wilcoxon test either with or without a block factor if mentioned in the text implemented in the R package “coin” v1.3-1^61^. The produced *p*-values were adjusted for multiple testing using the Benjamini–Hochberg false-discovery rate (of 5%, FDR) as reported in the text. The proportion of explained variance (R^2^) and significance of each clinical covariate was quantified by PERMANOVA calculated based on hamming distance of core genes or Jaccard distance of presence/absence of genes per genome, as implemented in the adonis2 function from R package “vegan” v2.5-7^62^ with 1000 permutations. Genomes with missing metadata for the given covariate were excluded.

### Reporting summary

Further information on research design is available in the Nature Research Reporting Summary linked to this article.

## Supplementary information

Supplementary Information

Description of Additional Supplementary Files

Supplementary Data 1

Supplementary Data 2

Supplementary Data 3

Supplementary Data 4

Supplementary Data 5

Supplementary Data 6

Supplementary Data 7

Supplementary Data 8

Reporting Summary

## Data Availability

The 32,277 genome assemblies, 2172 representatives of ELGG, and protein catalog of ELGP reported in this paper have been deposited in the Zenodo repository under 10.5281/zenodo.6969520. The other data supporting the findings of this study are available within the paper and additional files. Source data are provided with this paper.

## Source data

Source Data

## Code availability

All mentioned tools used for the data analysis in this study are publicly available, and the version and parameters used have been indicated. No custom code or pipeline was generated in this manuscript.
