# Supplementary material for: A compendium of 32,277 metagenome-assembled genomes and over 80 million genes from the early-life human gut microbiome
Source: Nat Commun. 2022 Sep 1;13:5139. doi: 10.1038/s41467-022-32805-z (PMC9437082; doi:10.1038/s41467-022-32805-z)
Supplement: Supplementary file 1 — Supplementary Information [file 41467_2022_32805_MOESM1_ESM.pdf]

Supplementary Information: A compendium of 32,277 metagenome-assembled genomes and over 80 million genes from the early-life human gut microbiome

S. Wang et al.

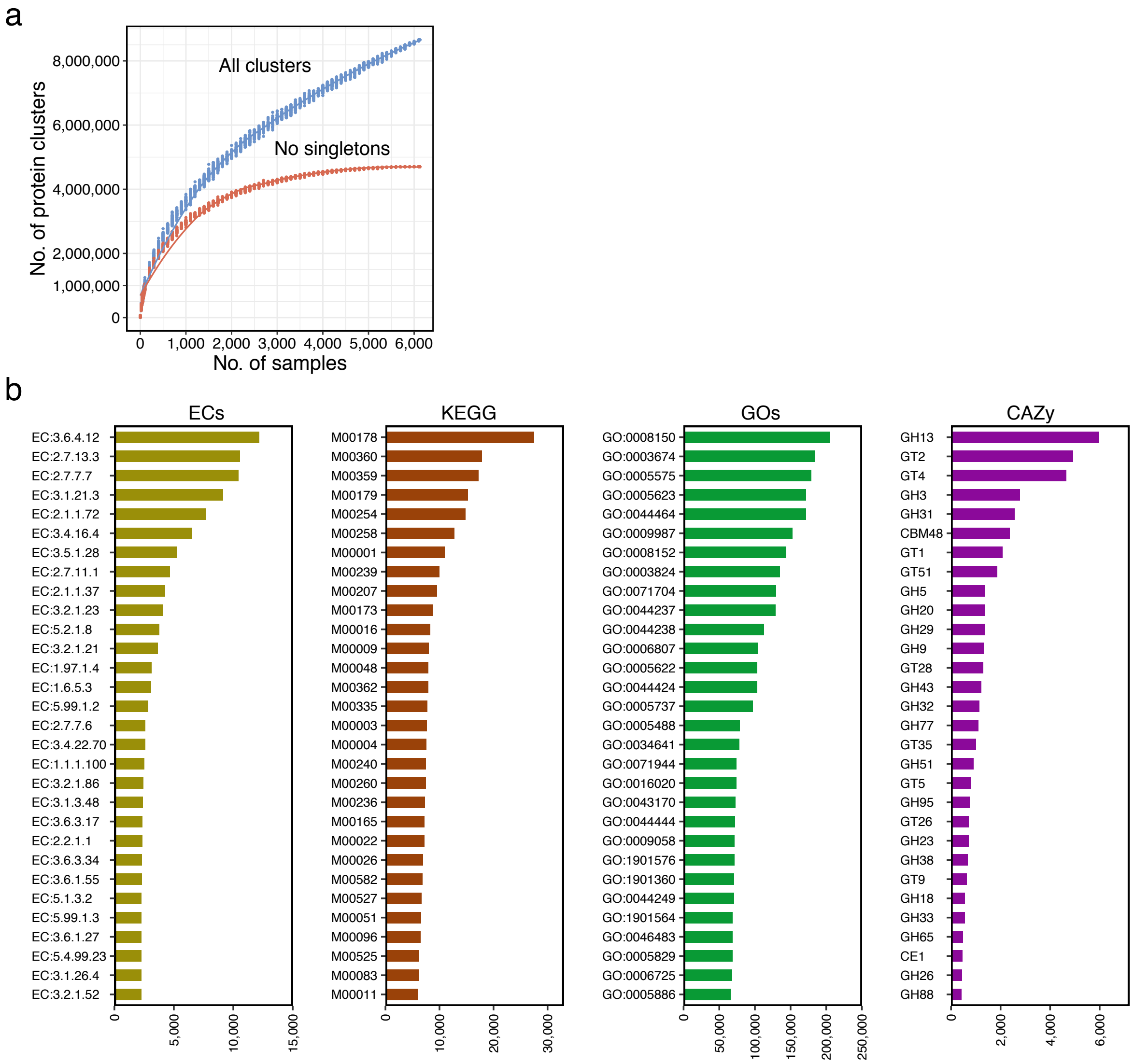

Supplementary Fig. 1 The rarefaction analysis and top functional families of ELGP catalog. a, Rarefaction analysis of the number of protein clusters of the early-life gut microbiome at 95% amino acid identity as a function of the number of samples included. Curves are depicted for all the protein clusters and after excluding singleton protein clusters (containing only one protein sequence). **b**, The top 30 highly represented functional families of ECs, KEGG, GOs, and CAZy from ELGP catalog.

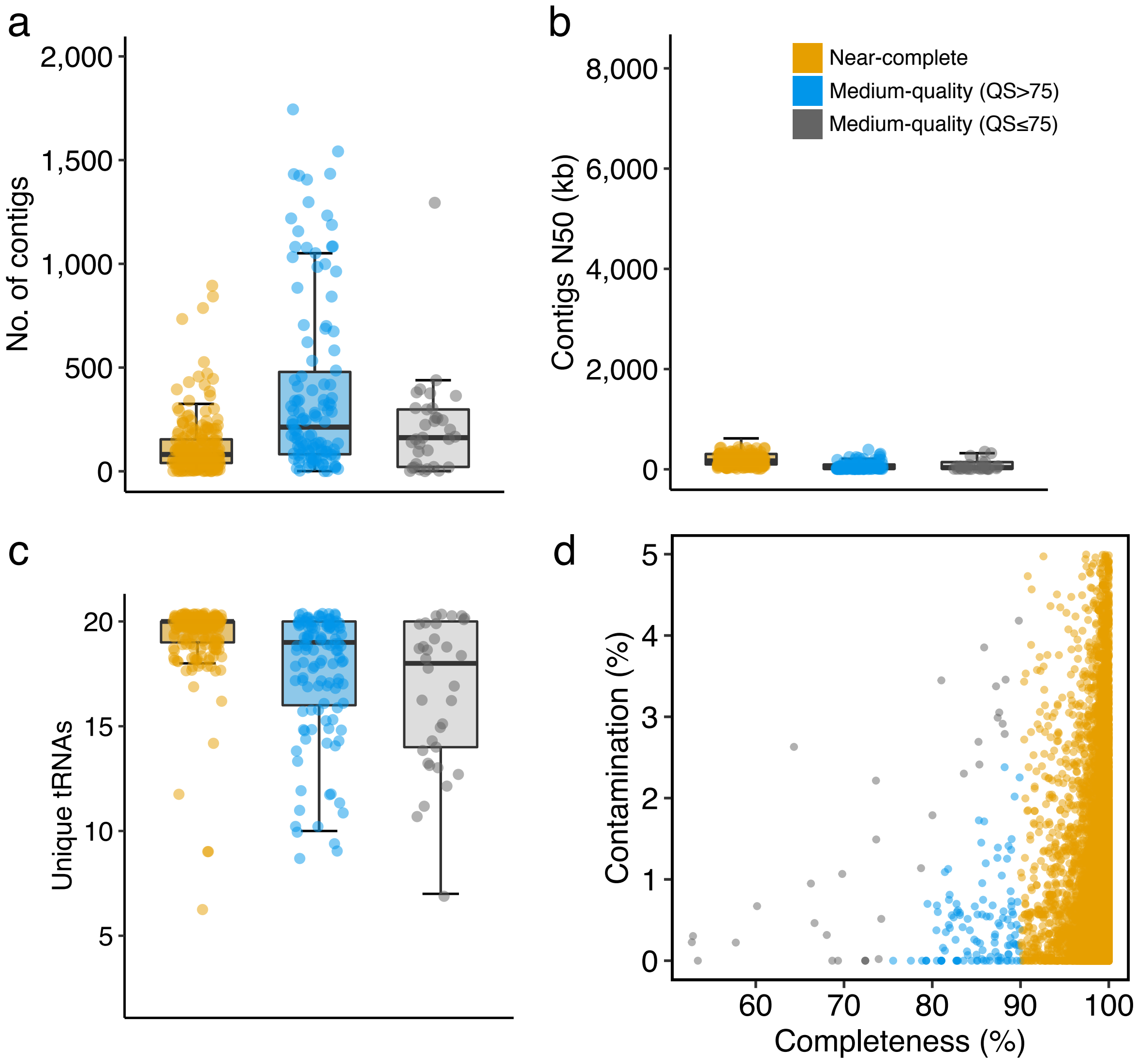

Supplementary Fig. 2 The quality metrics of reference genomes clustered with MAGs. **a**, The number of contigs of genomes of near-complete (n = 86,132), medium with quality score (QS) >75 (n = 118) and medium with QS ≤75 (n = 33) reference genomes. **b**, Contigs N50 of near-complete (n = 86,132), medium with quality score (QS) >75 (n = 118) and medium with QS ≤75 (n = 33) reference genomes. **c**, The number of tRNA of 86,283 of near-complete (n = 86,132), medium with quality score (QS) >75 (n = 118) and medium with QS ≤75 (n = 33) reference genomes. The boxes in **a**, **b**, and **c** show the interquartile range (IQR), with the horizonal line as the median, the whiskers indicating the range of the data (up to 1.5× IQR), and points beyond the whiskers as outliers. **d**, Completeness and contamination scores for each of 86,283 genomes. QS = completeness–5×contamination.

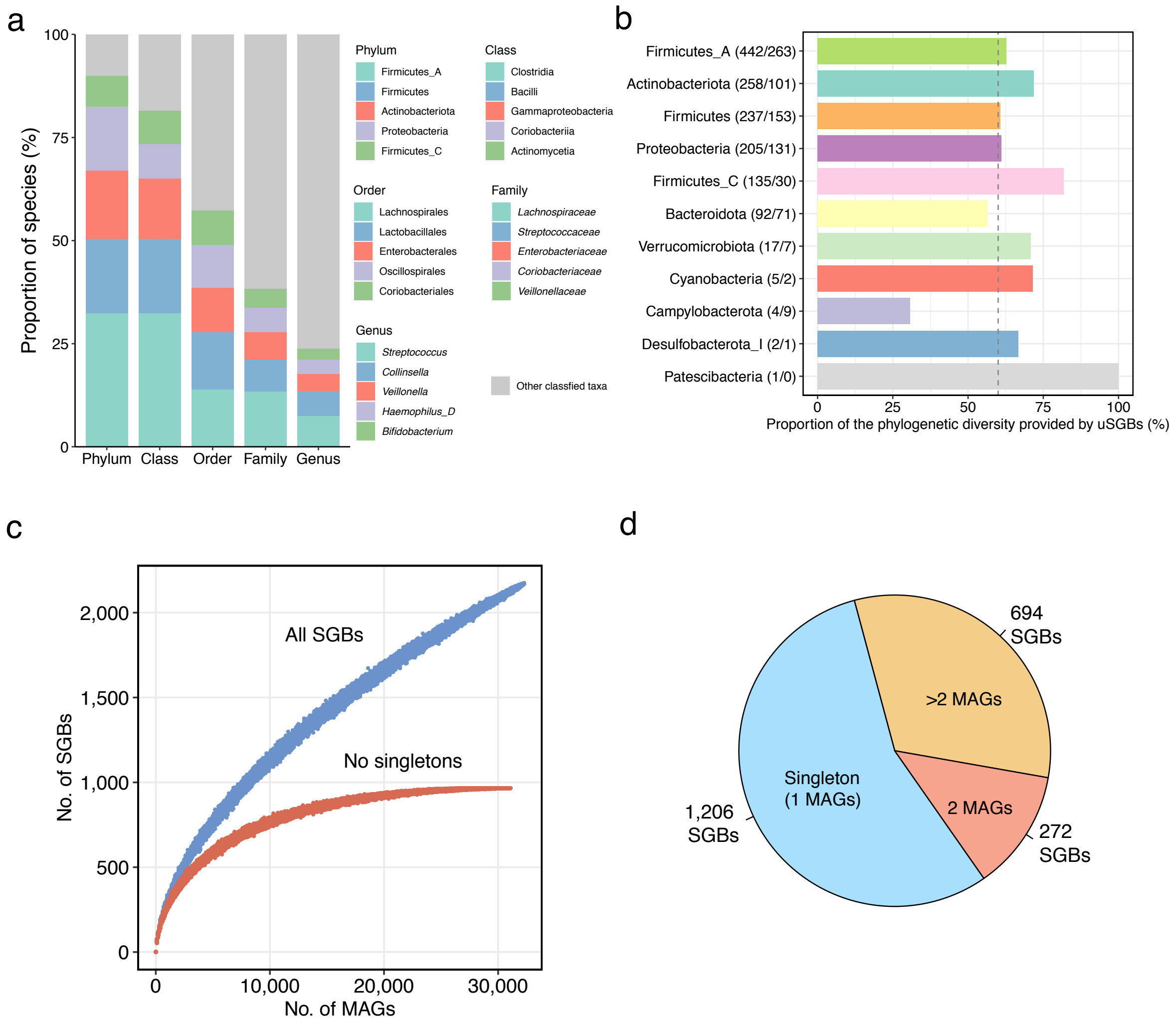

Supplementary Fig. 3 Taxonomic profile of the early-life human gut microbiome. a, The top five taxa at different resolutions of 2,172 species-level clusters (SGBs). **b**, The proportion of uSGBs in the total SGBs belonging to the phylum. The number in parentheses indicate the number of uSGBs and cSGBs in each phylum, respectively. **c**, Rarefaction analysis of the number of SGBs as a function of the number of MAGs included. Curves are depicted both for all the SGBs (blue) and after excluding singleton SGBs (represented by only one MAG; orange). **d**, A large proportion of the 1,206 newly reconstructed SGBs were represented by only a single genome.

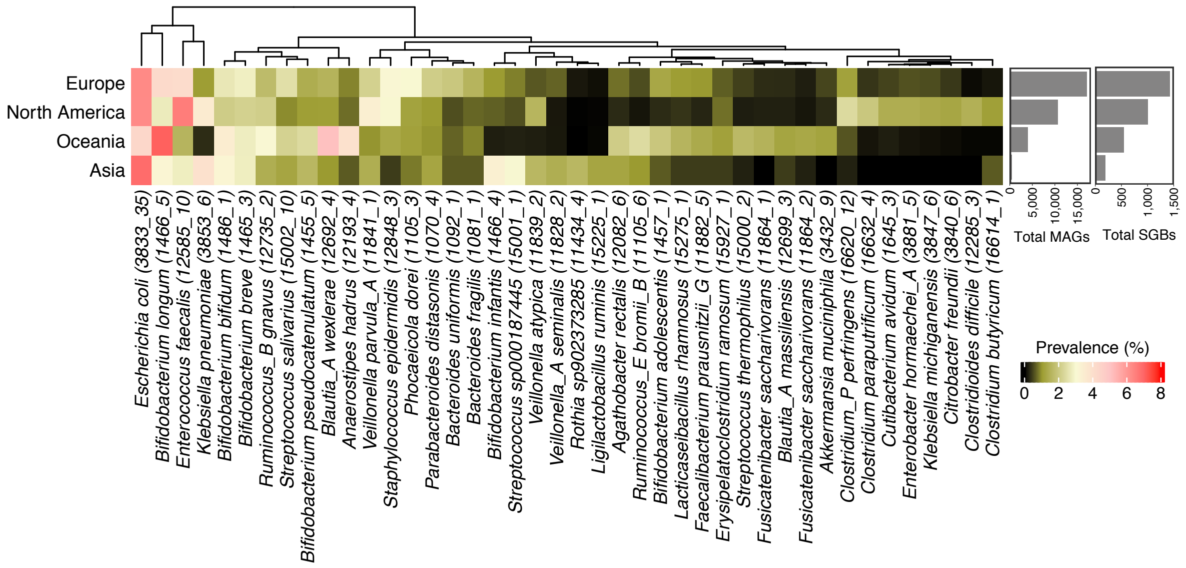

Supplementary Fig. 4 The geographic prevalence of species in each continent. Only the species with at least 1% prevalence in any of continents were plotted. The number in parentheses after each species indicates the SGBs ID. The bar on the right shows the total number of MAGs and total number of species observed in each continent, respectively.

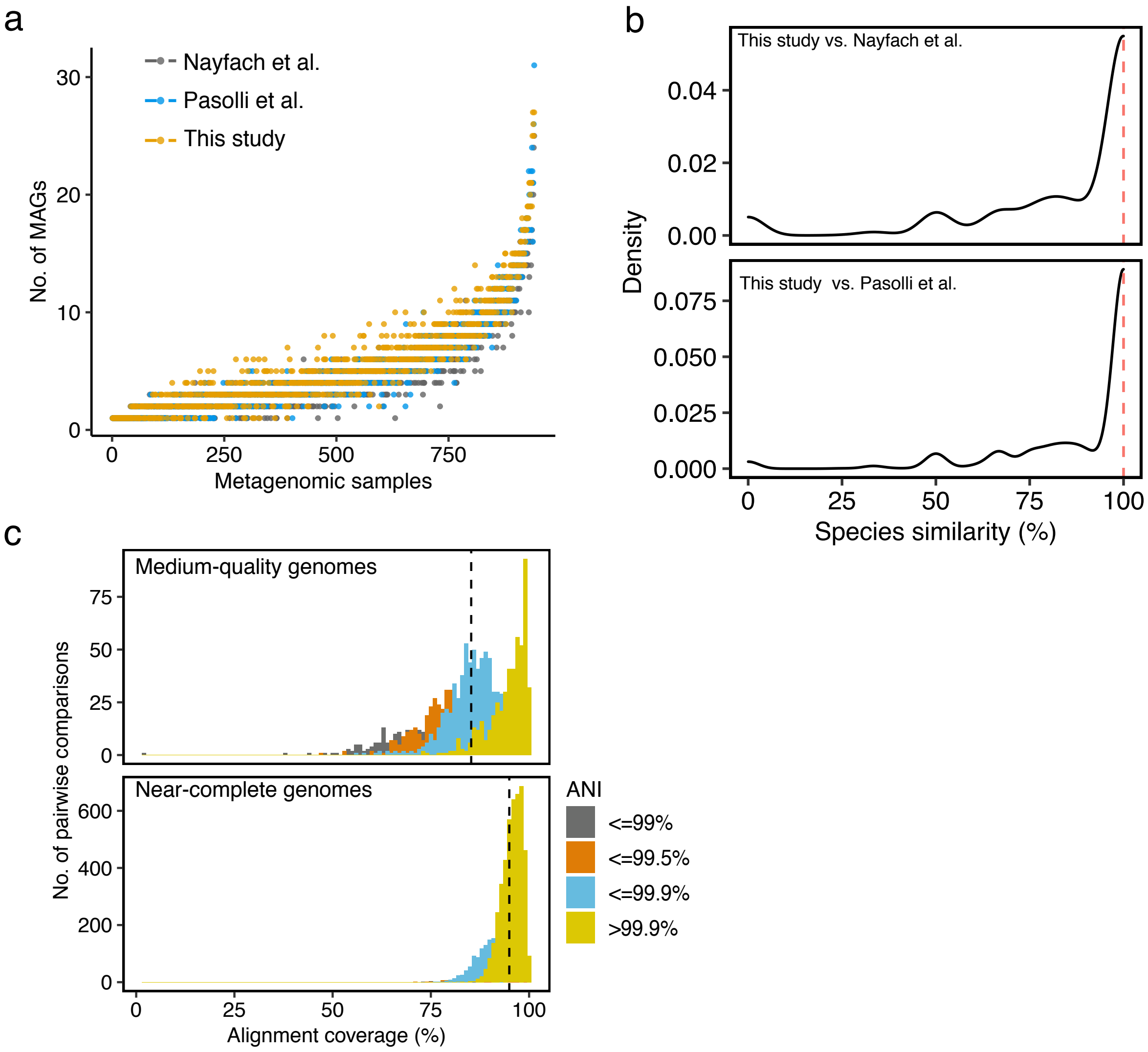

Supplementary Fig. 5 Comparisons of SGBs across three MAG studies with the same fecal metagenomes. a, The number of MAGs reconstructed by each study and the samples were ordered by the number of MAGs. **b**, The density plot depicted the similarity of SGBs from the MAGs reconstructed by different MAG studies, i.e., comparisons between this study to Nayfach et al. (top) and Pasolli et al. (bottom). **c**, The bar depicted the comparisons regarding estimated aligned fractions and average nucleotide identities (ANI) between conspecific MAGs obtained from the same fecal metagenomes by different MAG studies. The top panel shows results for medium-quality MAGs (>50% completeness and <5% contamination), and the bottom panel for near-complete (>90% completeness and <5% contamination) MAGs. Vertical dashed lines in Supplementary Fig. b and c denote the median values.

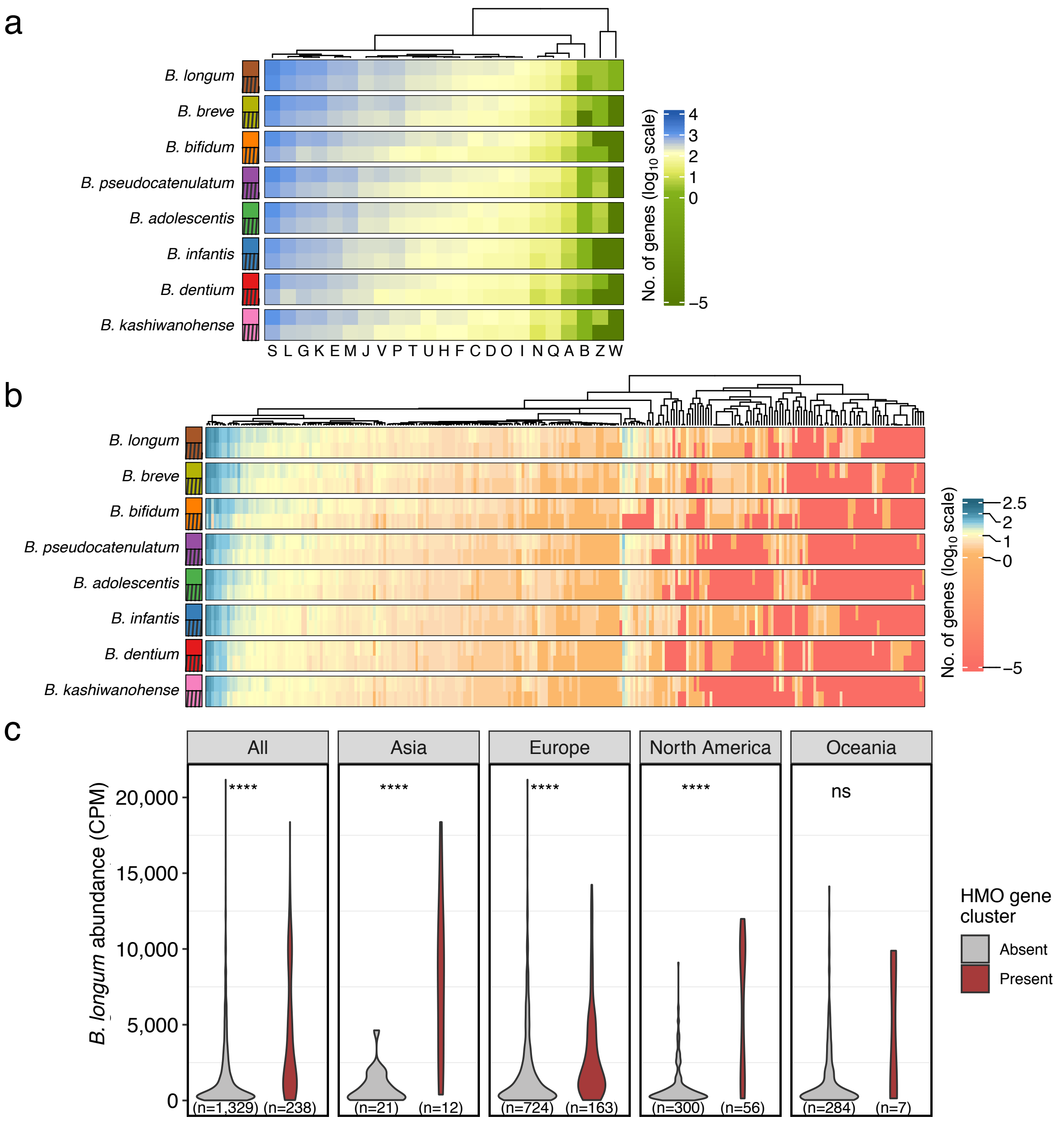

Supplementary Fig. 6 The functional annotation of *Bifidobacterium* spp. from ELGG catalog based on COGs and KEGG module databases. a, The functional annotations (columns) of the genome of each species (rows) based on COGs database. **b**, A total of 271 KEGG modules (columns) were encoded by genes (rows) from those eight bifidobacterial species. **c**, The relative abundance of *B. longum* strains stratified by continents and the presence of HMO gene cluster of *B. longum* strain. The strains of *B. infantis* (n = 236) and *B. longum* (n = 2) (found in Fig. 4e containing at least 15 HMO gene homologues) had higher relative abundance compared to other *B. longum strains* on average, even stratified by continents. The number of strains in parentheses is indicated below each box for all and continents. CPM, copies per million reads. ****$p < 0.0001$, ns: $p > 0.05$ based on two-tailed Wilcoxon test.

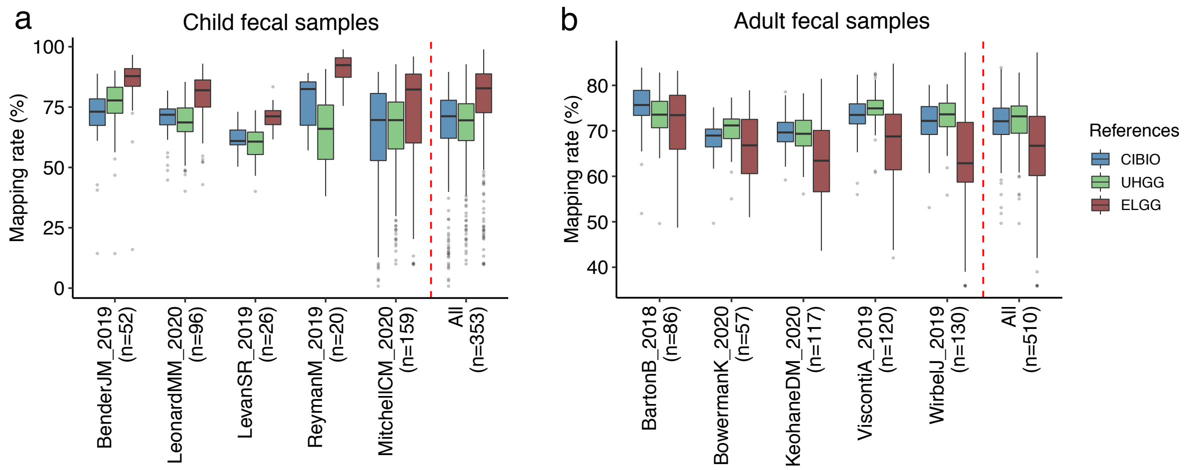

Supplementary Fig. 7 Over 80% representation of children's gut microbiome by ELGG genomes. **a,** Comparisons of mapping rate between references genomes from ELGG (n = 2,172), CIBIO (n = 4,930), and UHGG (n = 4,644) with 353 fecal metagenomes from children. **b,** Comparisons of mapping rate between references genomes from ELGG (n = 2,172), CIBIO (n = 4,930), and UHGG (n = 4,644) with 510 fecal metagenomes from adults. The boxes show the interquartile range (IQR), with the horizonal line as the median, the whiskers indicating the range of the data (up to 1.5× IQR), and points beyond the whiskers as outliers. The number in parentheses indicates the number of fecal metagenomes.

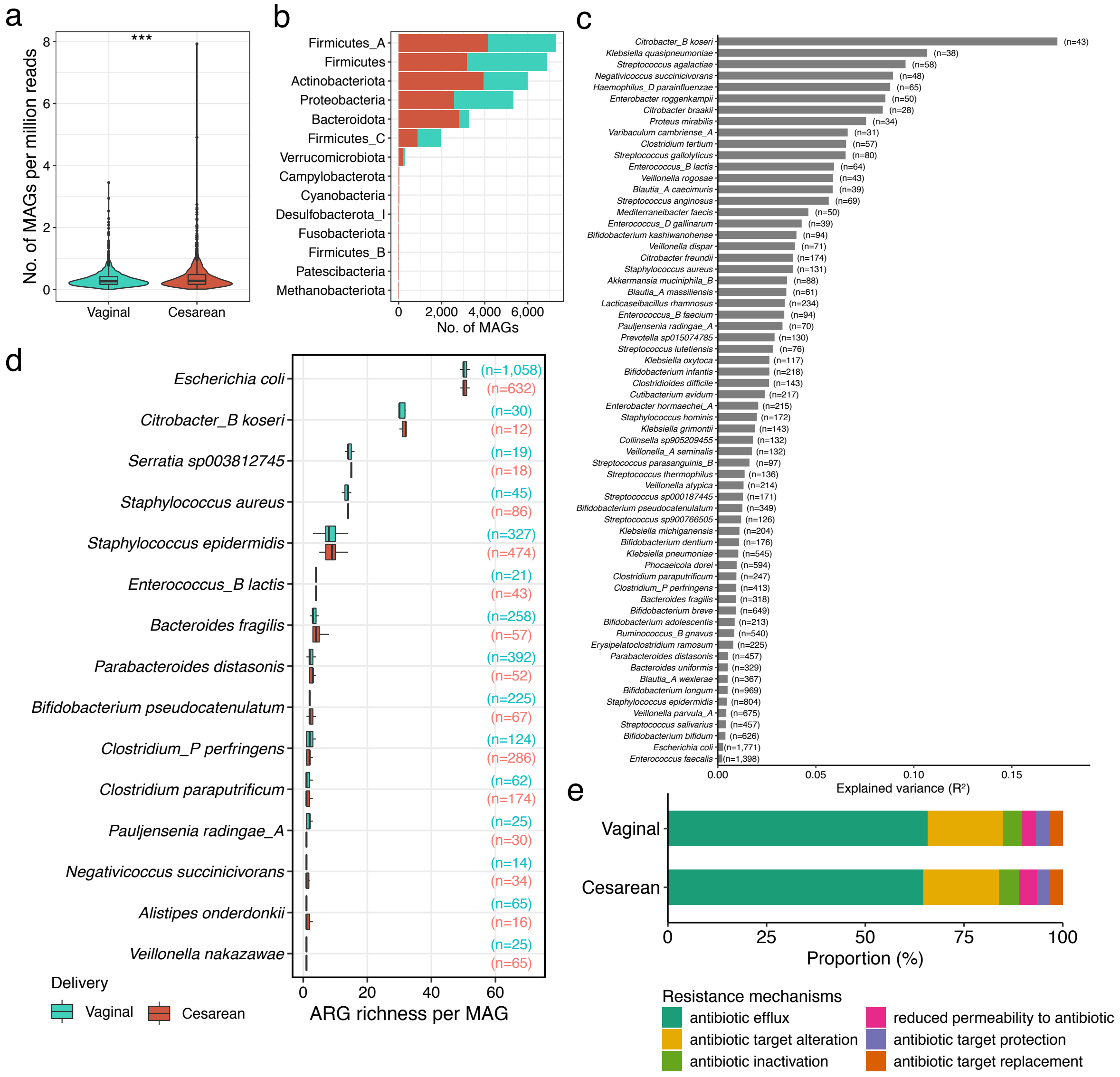

Supplementary Fig. 8 Conspecific genomic diversity stratified by delivery mode. a, The number of MAGs per million reads reconstructed from fecal metagenomes from children born by C-section or vaginally (two-tailed Wilcoxon test, ***p < 0.001). The boxes show the interquartile range (IQR), with the horizonal line as the median, the whiskers indicating the range of the data (up to 1.5× IQR), and points beyond the whiskers as outliers. **b**, The taxa assignment at phylum level of MAGs from children born by C-section or vaginally. **c**, The explained variance (R^2) contributed by delivery mode of 64 species that were significantly (PERMANOVA, FDR < 0.05) associated with delivery mode based on the Jaccard distance of gene presence/absence within each species. The number in parentheses indicated the number of MAGs of this species. **d**, A total of 15 species stratified by delivery mode contained significant (two-tailed Wilcoxon test, p < 0.05) differences in term of the richness of ARGs of MAGs. The boxes show the IQR, with the vertical line as the median and the whiskers indicating the range of the data (up to 1.5× IQR). The number in parentheses indicates the number of MAGs. **e**, Mechanisms of resistance of each ARG, depicted as a proportion of all ARGs detected in the species analyzed.

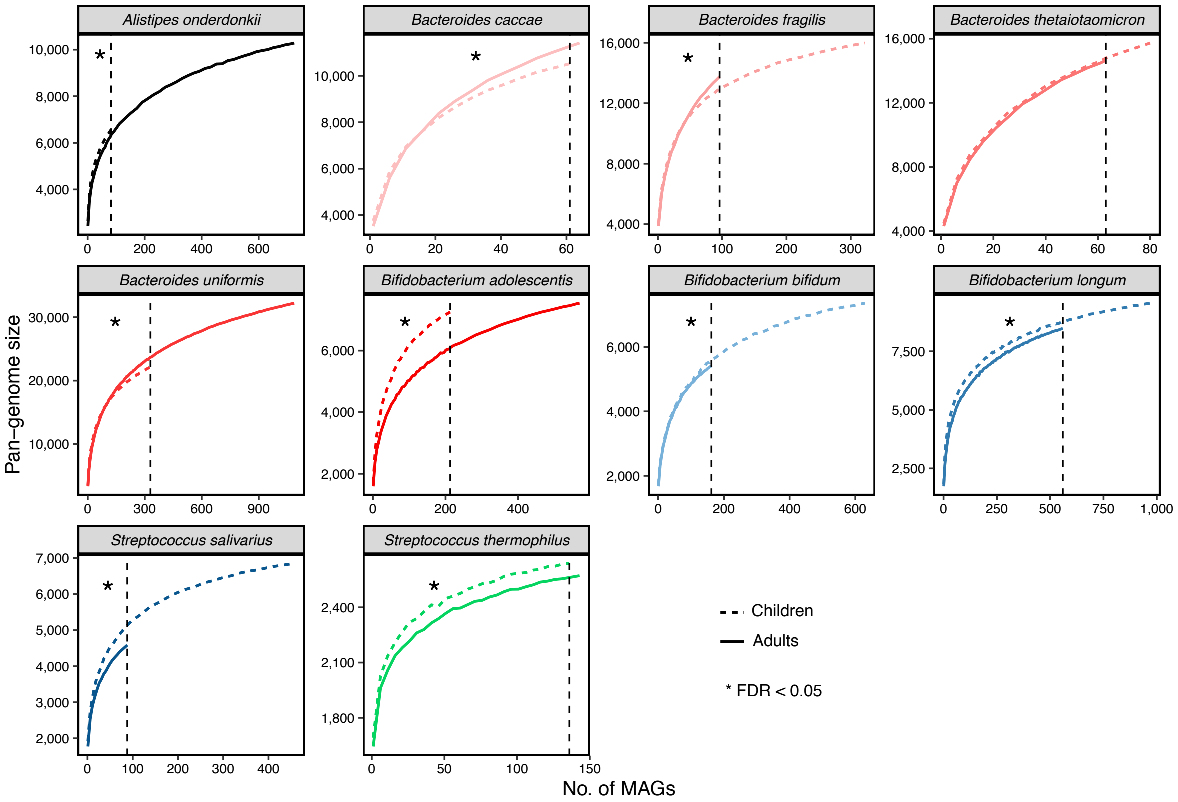

Supplementary Fig. 9 Pan-genome plot represented by the accumulated number of genes against the number of genomes of 10 species stratified by children and adults (two-tailed Wilcoxon test, *FDR < 0.05).

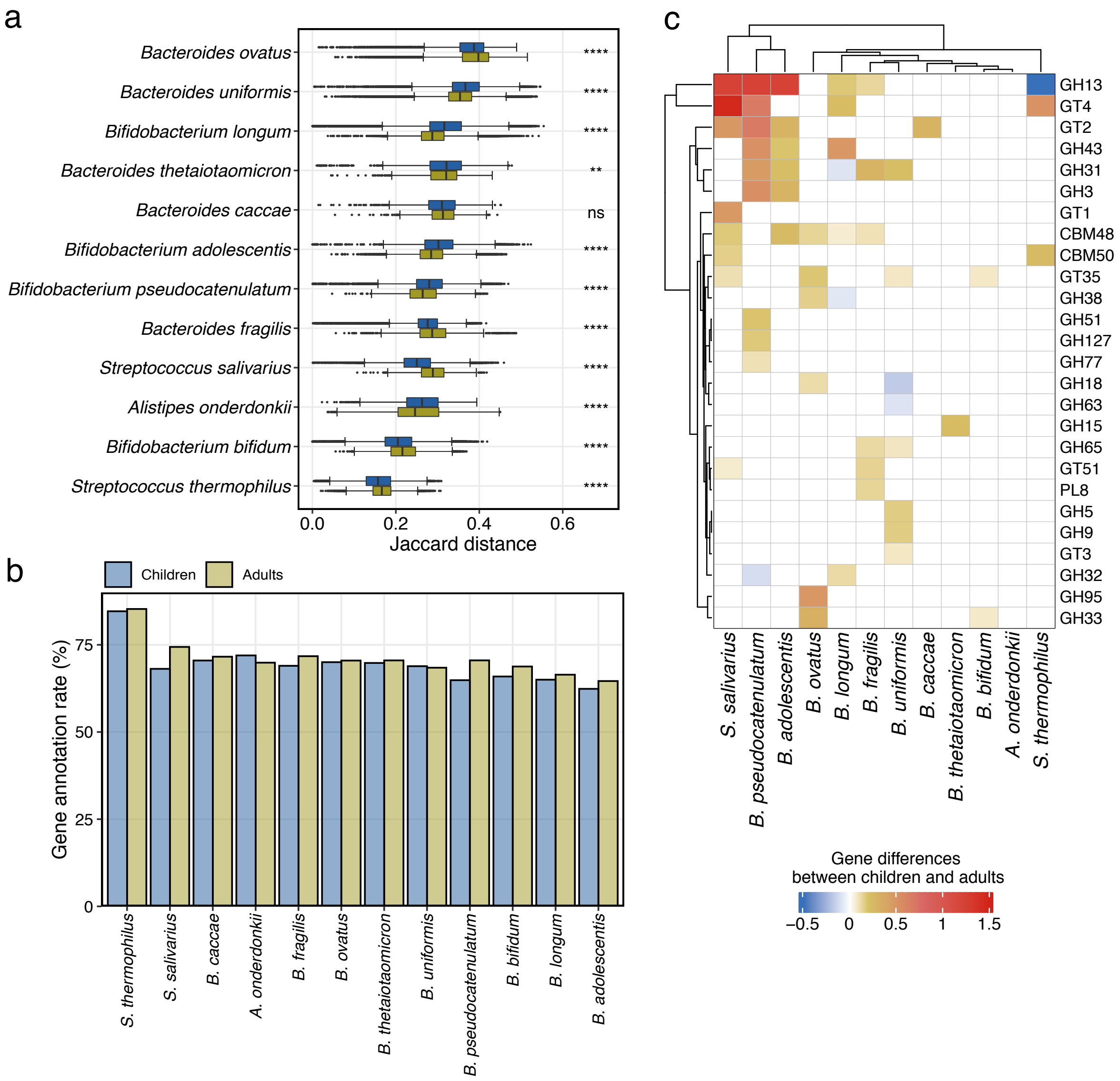

Supplementary Fig. 10 Comparison of gut microbiome between children and adults. a, Differences of gene number per genomes of each species stratified by delivery mode (left panel), as well as differences of Jaccard distance based on the presence/absence genes per genome (right panel). **FDR < 0.01, ****FDR < 0.0001 based on two-tailed Wilcoxon test. The boxes show the interquartile range (IQR), with the vertical line as the median, the whiskers indicating the range of the data (up to 1.5× IQR), and points beyond the whiskers as outliers. **b**, The rate of functional annotation across databases of COGs, KEGG, GOs, ECs, and CAZy for each species from children and adults. **c**, Functional annotated with CAZy database for the differential genes (Wilcoxon test, FDR < 0.05) from genomes between children and adults for each species.
