# Supplementary material for: A compendium of 32,277 metagenome-assembled genomes and over 80 million genes from the early-life human gut microbiome
Source: Nat Commun. 2022 Sep 1;13:5139. doi: 10.1038/s41467-022-32805-z (PMC9437082; doi:10.1038/s41467-022-32805-z)
Supplement: Supplementary file 2 — Description of Additional Supplementary Files [file 41467_2022_32805_MOESM2_ESM.doc]

**Supplementary Data legends**

**Supplementary Data 1.** Description of the 26 datasets and 6,122 fecal metagenomes from the human early life used in this study

**Supplementary Data 2.** Assembly statistics, cluster identity, and taxonomic lineage of the 32,277 early-life human gut microbial genomes used to generate the ELGG catalog

**Supplementary Data 3.** Assembly statistics, cluster identity, and taxonomic lineage of the 2,172 representatives of ELGG catalog

**Supplementary Data 4.** Genomes from this study (ELGG, n = 5,203), Nayfach et al. (n = 4,284) and Pasolli et al. (n = 4,728) to investigate the reproducibility of SGBs from ELGG catalog

**Supplementary Data 5.** Number of genes from genomes belonging to each bifidobacterial species assigned to 271 KEGG modules

**Supplementary Data 6.** The mapping rate of 353 fecal metagenomes from children and 510 fecal metagenomes from adults against the representative genomes of ELGG (n = 2,172), CIBIO (n = 4,930) and UHGG (n = 4,644)

**Supplementary Data 7.** Statistics of genomes used to compare genomic differences from children born by C-section or vaginally

**Supplementary Data 8.** Unique functional families belonging to genomes of children and adults annotated by COGs, KEGG, GOs, ECs, and CAZy databases
