# Supplementary material for: A compendium of 32,277 metagenome-assembled genomes and over 80 million genes from the early-life human gut microbiome
Source: Nat Commun. 2022 Sep 1;13:5139. doi: 10.1038/s41467-022-32805-z (PMC9437082; doi:10.1038/s41467-022-32805-z)
Supplement: Supplementary file 11 — Reporting Summary [file 41467_2022_32805_MOESM11_ESM.pdf]

nature portfolio

Corresponding author(s): Shaopu Wang

Last updated by author(s): Aug 9, 2022

Reporting Summary

Nature Portfolio wishes to improve the reproducibility of the work that we publish. This form provides structure for consistency and transparency in reporting. For further information on Nature Portfolio policies, see our Editorial Policies and the Editorial Policy Checklist.

Statistics

For all statistical analyses, confirm that the following items are present in the figure legend, table legend, main text, or Methods section.

n/a	Confirmed	
☐	☒	The exact sample size (n) for each experimental group/condition, given as a discrete number and unit of measurement
☒	☐	A statement on whether measurements were taken from distinct samples or whether the same sample was measured repeatedly
☐	☒	The statistical test(s) used AND whether they are one- or two-sided<br>*Only common tests should be described solely by name; describe more complex techniques in the Methods section.*
☐	☒	A description of all covariates tested
☐	☒	A description of any assumptions or corrections, such as tests of normality and adjustment for multiple comparisons
☐	☒	A full description of the statistical parameters including central tendency (e.g. means) or other basic estimates (e.g. regression coefficient) AND variation (e.g. standard deviation) or associated estimates of uncertainty (e.g. confidence intervals)
☐	☒	For null hypothesis testing, the test statistic (e.g. F, t, r) with confidence intervals, effect sizes, degrees of freedom and P value noted<br>*Give P values as exact values whenever suitable.*
☒	☐	For Bayesian analysis, information on the choice of priors and Markov chain Monte Carlo settings
☒	☐	For hierarchical and complex designs, identification of the appropriate level for tests and full reporting of outcomes
☐	☒	Estimates of effect sizes (e.g. Cohen's d, Pearson's r), indicating how they were calculated

Our web collection on statistics for biologists contains articles on many of the points above.

Software and code

Policy information about availability of computer code

Data collection	A total of 6,122 paired-end sequencing runs were downloaded from the NCBI SRA according to the accession numbers published in each included study and then converted into FASTQ with SRA Toolkit v2.9.1 (fastq-dump v2.9.1). The 353 additional paired-end sequencing runs from children and 510 paired-end sequencing runs from adults from metagenomic read mapping analysis were downloaded from the NCBI SRA according to the accession numbers published in each included study and then converted into FASTQ with SRA Toolkit v2.11.2.
Data analysis	KneadData v0.7.2; MegaHIT v1.1.3; BBMap v38.22; BWA-MEM v0.7.17; SAMtools v1.10; MetaBAT v2.12.1; MaxBin v2.2.6; CONCOCT v1.0.0; metaWRAP v1.3.1; CheckM v1.0.12; GUNC v1.0.5; Barrnap v0.9; tRNAScan-SE v2.0.6; CMseq tool v1.0.3; Prodigal v2.6.3; Prokka v1.14.6; dRep v2.6.2; fastANI v1.33; Mash v2.3; GTDB-Tk v2.1.0; FastTree v2.1.11; Bowtie2 v2.4.5; CD-HIT v4.8.1; Panaroo v1.2.10; eggNOG-mapper v2.1.7; RGI, v5.2.0; Blast v2.9.0; additional R packages used have been given in Methods, including coin v1.3-1, vegan v2.5-7.

For manuscripts utilizing custom algorithms or software that are central to the research but not yet described in published literature, software must be made available to editors and reviewers. We strongly encourage code deposition in a community repository (e.g. GitHub). See the Nature Portfolio guidelines for submitting code & software for further information.

Data

Policy information about availability of data

All manuscripts must include a data availability statement. This statement should provide the following information, where applicable:

- Accession codes, unique identifiers, or web links for publicly available datasets
- A description of any restrictions on data availability
- For clinical datasets or third party data, please ensure that the statement adheres to our policy

The gut metagenomic data were downloaded from the public database based on the accession number of included studies. The reference genomes were downloaded from NCBI RefSeq database, Human Gastrointestinal Bacteria Culture Collection (HBC, http://ftp.ebi.ac.uk/pub/databases/metagenomics/genome_sets/hbc_genomes.tar.gz), and Culturable Genome Reference (CGR). The UHGG representative genomes and adult genomes were then downloaded from the MGnify FTP site (http://ftp.ebi.ac.uk/pub/databases/metagenomics/mgnify_genomes/). The hg19 human reference genome for removing human contamination was downloaded and installed by following the instruction of KneadData.

The 32,277 genome assemblies, 2,172 representatives of ELGG, and protein catalog of ELGP reported in this paper have been deposited in the Zenodo repository under https://doi.org/10.5281/zenodo.6969520. The other data supporting the findings of this study are available within the paper and additional files. Source data are provided with this paper.

Human research participants

Policy information about studies involving human research participants and Sex and Gender in Research.

Reporting on sex and gender	No recruitment occurred in this study, and the gender information (2,818 female and 2,878 male) was collected based on the publicly available metadata of each study. The gender information was just used to give the overall description of 6,122 samples, and not used for any other data analyses.
Population characteristics	The 6,122 samples from children under three years old were distributed among 11 countries across four continents, with the United States, United Kingdom, and New Zealand being the top three represented.
Recruitment	No recruitment occurred as this study is a meta-analysis with the publicly available shotgun metagenomics data.
Ethics oversight	Not applicable as this study is a meta-analysis with the publicly available shotgun metagenomics data.

Note that full information on the approval of the study protocol must also be provided in the manuscript.

Field-specific reporting

Please select the one below that is the best fit for your research. If you are not sure, read the appropriate sections before making your selection.

☒ Life sciences ☐ Behavioural & social sciences ☐ Ecological, evolutionary & environmental sciences

For a reference copy of the document with all sections, see nature.com/documents/nr-reporting-summary-flat.pdf

Life sciences study design

All studies must disclose on these points even when the disclosure is negative.

Sample size	No statistical method was used to predetermine the sample size, and this meta-analysis exclusively included a total of 6,122 publicly available early-life human gut metagenome samples that were published with detailed metadata.
Data exclusions	Genomes were filtered to have completeness >50% and contamination <5% together with genome quality score (defined as completeness–5×contamination) >50 and free of chimerism.
Replication	To investigate the reproducibility of species-level clusters in current study, we compared a common set of 941 gut metagenomes used in current study and another two studies with different assembly and binning approaches. The results suggested a high reproducibility of popular assembly and binning tools in large-scale genome reconstructions used in current study.
Randomization	Randomization was not applicable as we analyzed publicly available data without experiments.
Blinding	Blinding was not applicable as we analyzed publicly available data with no allocation to hide.

Reporting for specific materials, systems and methods

We require information from authors about some types of materials, experimental systems and methods used in many studies. Here, indicate whether each material, system or method listed is relevant to your study. If you are not sure if a list item applies to your research, read the appropriate section before selecting a response.

Materials & experimental systems

n/a	Involved in the study
☒	☐ Antibodies
☒	☐ Eukaryotic cell lines
☒	☐ Palaeontology and archaeology
☒	☐ Animals and other organisms
☒	☐ Clinical data
☒	☐ Dual use research of concern

Methods

n/a	Involved in the study
☒	☐ ChIP-seq
☒	☐ Flow cytometry
☒	☐ MRI-based neuroimaging
